# JARID2 promotes invasion and metastasis of hepatocellular carcinoma by facilitating epithelial-mesenchymal transition through PTEN/AKT signaling

**DOI:** 10.18632/oncotarget.9733

**Published:** 2016-05-31

**Authors:** Xiong Lei, Jiang-Feng Xu, Rui-Min Chang, Feng Fang, Chao-Hui Zuo, Lian-Yue Yang

**Affiliations:** ^1^ Liver Cancer Laboratory, Xiangya Hospital, Central South University, Changsha 410008, Hunan, China; ^2^ Department of Surgery, Xiangya Hospital, Central South University, Changsha 410008, Hunan, China; ^3^ Department of Abdominal Surgical Oncology, Affiliated Cancer Hospital of Xiangya School of Medicine, Central South University, Changsha 410013, Hunan, China

**Keywords:** JARID2, hepatocellular carcinoma, invasion, metastasis, epithelial-mesenchymal transition

## Abstract

JARID2 is crucial for maintenance of pluripotency and differentiation of embryonic stem cells. However, little is known about the role of JARID2 in metastasis of hepatocellular carcinoma (HCC). This study found that JARID2 expression was significantly higher in HCC tissues than that in adjacent non-tumor liver tissues (ANLTs), and its expression level correlated with HCC metastasis. High JARID2 expression was significantly correlated with multiple tumor nodules, high Edmondson-Steiner grade, microvascular invasion, advanced TNM stage and advanced BCLC stage (all *P* < 0.05) and indicated poor prognosis of HCC in training and validation cohorts (all *P* < 0.05) totaling 182 patients. High JARID2 expression was an independent and significant risk factor for disease-free survival (DFS; *P* = 0.017) and overall survival (OS; *P* = 0.041) after curative liver resection in training cohort, and also validated as an independent and significant risk factor for DFS (*P* = 0.033) and OS (*P* = 0.031) in validation cohort. Moreover, down-regulation of JARID2 dramatically inhibited HCC cell migration, invasion, proliferation *in vitro* and metastasis *in vivo*, whereas overexpression of JARID2 significantly increased migration, invasion, proliferation *in vitro* and metastasis *in vivo*. Mechanistically, the data showed that JARID2 exerted its function by repressing PTEN expression through increasing H3K27 trimethylation (H3K27me3) at PTEN promoter region, which subsequently resulted in activation of protein kinase B (AKT) and enhanced epithelial-mesenchymal transition (EMT). In conclusion, this study revealed that JARID2 promotes invasion and metastasis of HCC by facilitating EMT through PTEN/AKT signaling.

## INTRODUCTION

Hepatocellular carcinoma (HCC) is one of the most common cancers and ranks the second leading cause of cancer death in men and sixth in women over the world [1]. Liver resection is still considered as the prime choice and the most effective “curative” treatment for HCC, especially for solitary large HCC [2]. Despite the improvement in treatment of HCC during recent decades, the overall survival of patients with HCC remains unsatisfactory due to high rate of recurrence and metastasis after liver resection [2, 3]. Thus, it is essential to explore effective biomarkers for recurrence and metastasis to improve treatment strategy for better clinical outcome. Tumor metastasis is a complex process involving a complicate succession of invasion-metastasis steps and associated with numerous molecular genetic mechanisms. Though several molecules associated with metastasis of HCC had been found by our research group and others, [4–8] the mechanisms underlying HCC metastasis are still elusive. Recently, some researches have illustrated that the epithelial-mesenchymal transition (EMT) is an important mechanism promoting tumor metastasis, including HCC [9, 10]. EMT, a normal embryological process, is characterized by the loss of epithelial markers and the gain of mesenchymal markers [9]. HCC cells undergoing EMT acquire enhanced migratory and invasive properties, thereby resulting in HCC metastasis [11]. Therefore, it is necessary to elucidate the new regulatory mechanisms of EMT in HCC invasion and metastasis.

JARID2, one member of the Jumonji protein family, is crucial for maintenance of pluripotency and differentiation of embryonic stem cells [12, 13]. Its deletion was previously shown to result in severe defects in liver development [14]. Abnormal JARID2 expression had been reported in rhabdomyosarcomas and leukemia, [15, 16] and contributed to metastatic behavior of cancer cells by promoting epithelial-mesenchymal transition (EMT) [17]. JARID2 is also associated with leukemic transformation of chronic myeloid malignancies [16, 18]. These data suggest that JARID2 functions to the malignant character in tumors and also led us to hypothesize that JARID2 may play roles in HCC pathogenesis, especially in HCC metastasis. However, little is known about the function of JARID2 in the development of HCC. So we attempted to study the roles of JARID2 in the invasion and metastasis of human HCC.

Phosphatase and tension homolog (PTEN), a well-studied tumor suppressor phosphatase, plays a central role in regulating tumor growth and metastasis [19, 20]. It has been reported that PTEN mutation or deletion frequently occurs in various human cancers and is associated with cancer progression [20, 21]. However, increasing studies showed that downregulation of PTEN in HCC, rather than PTEN mutations or deletions, is believed to contribute to HCC metastasis, [11, 22] suggesting other mechanisms in HCC may be responsible for the downregulation of PTEN. Recently, it was shown downregulation of PTEN was also associated with trimethylation of H3 lysine 27 (H3K27me3) induced by polycomb repressive complex 2 (PRC2) [23]. JARID2 is essential for the binding of PRC2 to the targeted genes, leading to silence of gene expression by H3K27me3, [13] However, whether JARID2 could interact with the PRC2 complex to regulate PTEN expression in HCC remains unknown. Therefore, we also examined molecular mechanisms whether JARID2 exerted functions by regulating PTEN expression in HCC.

In this study, the data indicated that JARID2 was highly expressed in HCC tissues relative to the adjacent non-tumor liver tissues (ANLTs), which correlated with HCC metastasis and predicted poor prognosis of HCC patients. JARID2 promoted epithelial-mesenchymal transition (EMT)-mediated metastasis of HCC by suppressing PTEN and activating of AKT signaling.

## RESULTS

### JARID2 is significantly upregulated in HCC

**Figure 1:**
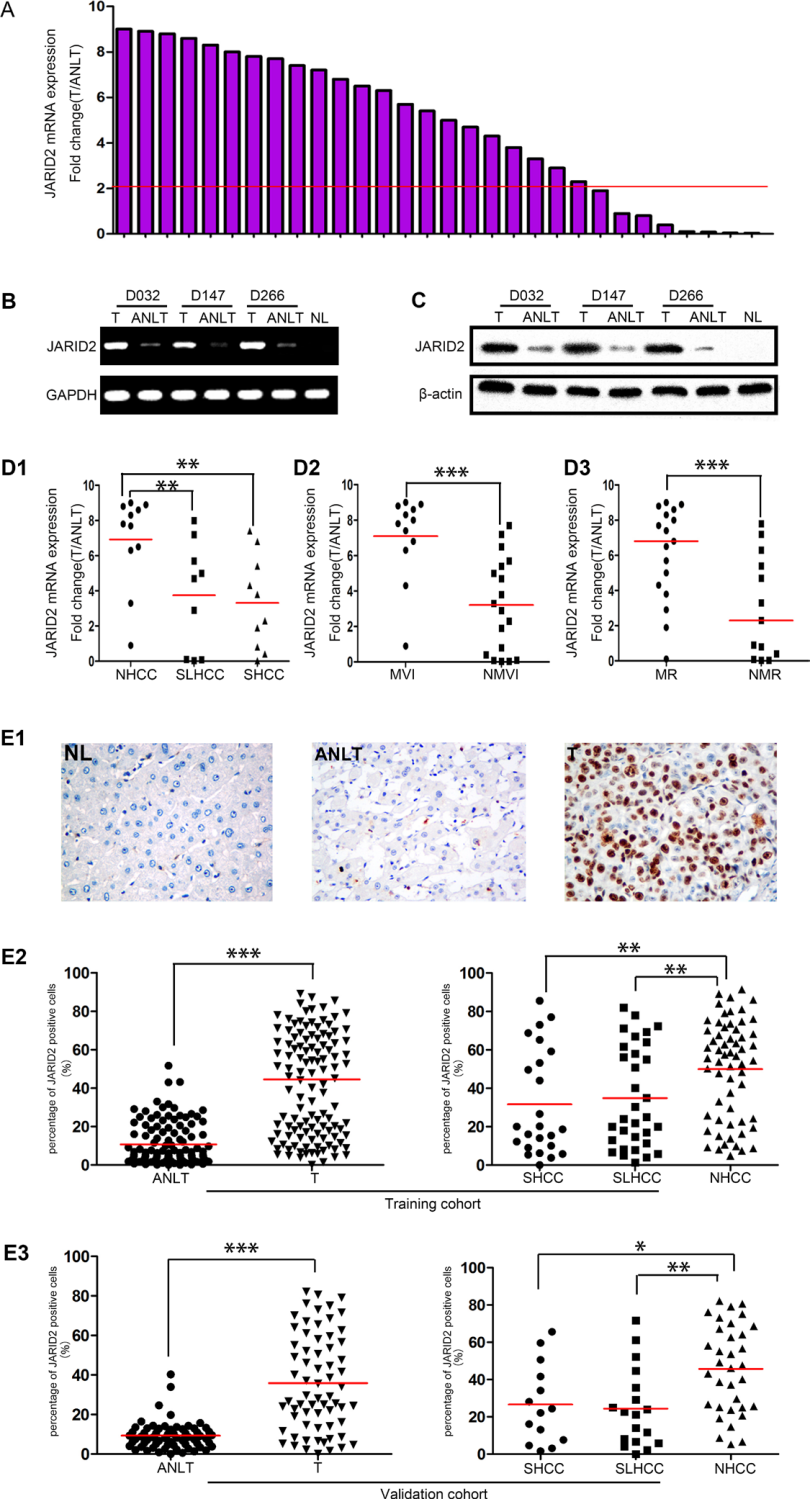
JARID2 is significantly upregulated in HCC tissues (**A**) qRT-PCR results showed that compared with matched ANLTs, the expression level of JARID2 was significantly up-regulated in HCC tissues. The value of JARID2 mRNA in ANLTs was used to normalize (value set to 1) the value of JARID2 mRNA in HCC tissues. Red line indicates fold change of JARID2 equal to 2. T, HCC tissue; ANLT, adjacent nontumorous liver tissue. (**B**) The representative images of semiquantitative RT-PCR showed that JARID2 mRNA in HCC tissues was significantly higher than those in ANLTs. (**C**) Western blot results showed that JARID2 protein in HCC tissues was significantly higher than those in ANLTs. (**D**) JARID2 mRNA expression level was detected by qRT-PCR in NHCC, SLHCC and SHCC. The expression of JARID2 in NHCC was significantly higher than in SLHCC or SHCC, but there was no significant difference between SLHCC and SHCC (**D1**). JARID2 mRNA expression level was detected by qRT-PCR in HCC tissues with MVI and those without MVI (**D2**) and in HCC tissues with metastasis and/or recurrence and those without metastasis and/or recurrence (**D3**). SHCC, small hepatocellular carcinoma; SLHCC, solitary large hepatocellular carcinoma; NHCC, nodular hepatocellular carcinoma; MVI, with microvascular invasion; NMVI, without microvascular invasion; MR, with metastasis and/or recurrence; NMR, without metastasis and/or recurrence. (**E**) JARID2 expression in training cohort (*n* = 116) and in validation cohort (*n* = 66). The JARID2 expression was determined by immunohistochemistry (IHC) and calculated by percent of positive cells. Representative IHC images of normal liver (NL), ANLT, HCC tissues (T) (**E1**). The expression of JARID2 in ANLTs, HCC tissues (T) and in HCC subtypes (SHCC, SLHCC and NHCC) in training cohort (**E2**) and validation cohort (**E3**). *P < 0.05; **P < 0.01; ***P < 0.001.

**Figure 2:**
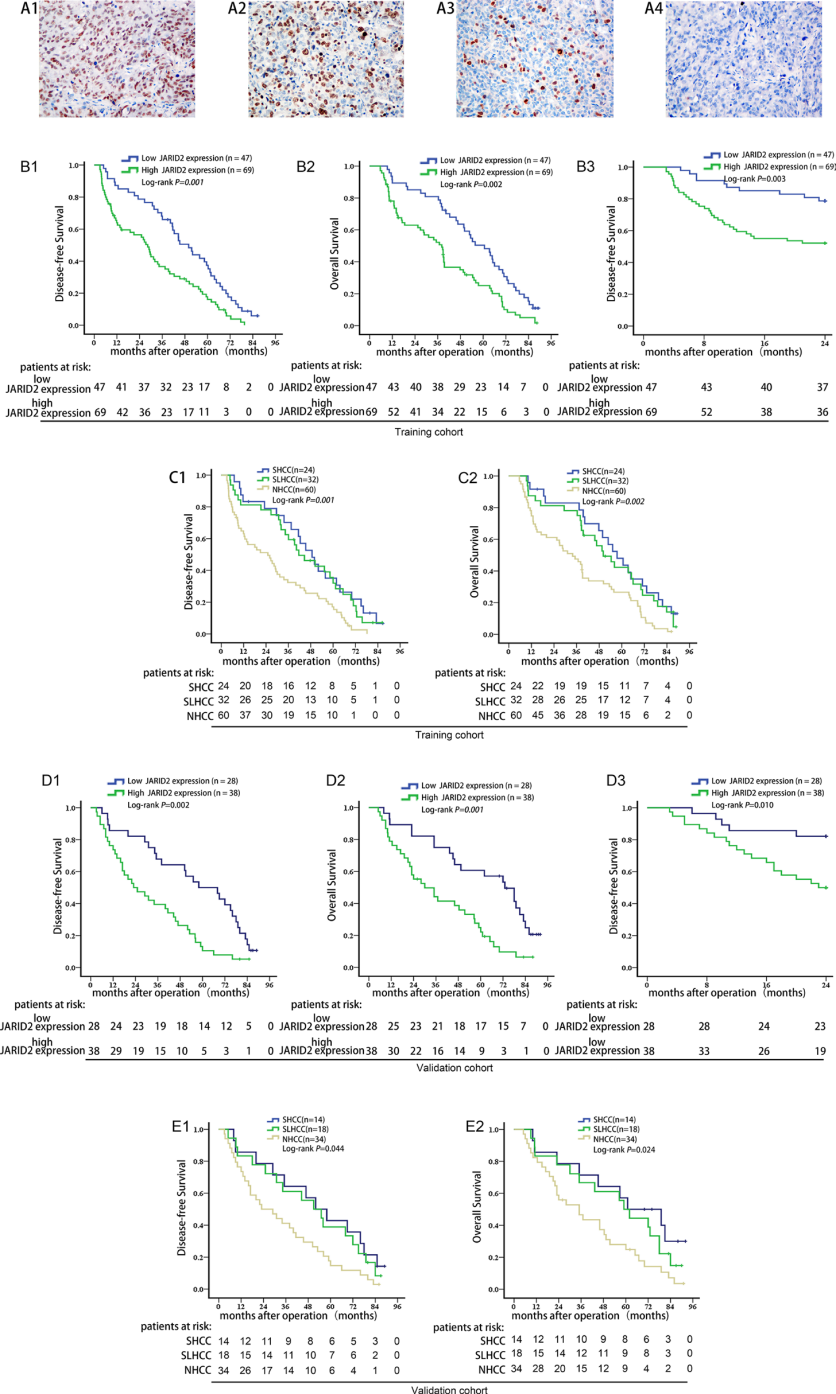
High JARID2 expression is significantly correlated with poor prognosis of HCC (**A**) Representative IHC images for JARID2 expression. A1, more than 51% of cancer cells (scored as 3+); A2, 26%–50% of cancer cells (scored as 2+); A3, 11%–25% of cancer cells (scored as 1+); A4, negative control. Original magnification: ×400. (**B**) Kaplan-Meier analysis of the correlation between JARID2 expression and disease-free survival (**B1**), overall survival (**B2**), early recurrence (**B3**) of HCC patients in training cohort. (**C**) Survival analysis of three subtypes of HCC in training cohort. (**D**) Kaplan-Meier survival analysis of the correlation between JARID2 expression and disease-free survival (**D1**), overall survival (**D2**), early recurrence (**D3**) in validation cohort. (**E**) Survival analysis of three subtypes of HCC in validation cohort.

First, quantitative real-time polymerase chain reaction (qRT-PCR) was used to detect JARID2 expression in 30 paired fresh HCC samples. The results showed that, compared with matched adjacent non-tumor liver tissues (ANLTs), the expression level of JARID2 in HCC tissues (T) was significantly up-regulated (fold change (T/ANLT) > 2) in 73.3% cases (22/30) (Figure 1A). RT-PCR (Figure 1B) and western blotting (Figure 1C) showed the similar results as qRT-PCR in these matched specimens. Interestingly, among three different subtypes of HCC including small hepatocellular carcinoma (SHCC, the diameter of HCC ≤ 5 cm), solitary large hepatocellular carcinoma (SLHCC, only one nodule, and diameter > 5 cm, and grows expansively within an intact capsule or pseudocapsule), nodular hepatocellular carcinoma (NHCC, has more than 2 nodules), [2, 24] JARID2 expression level in NHCC with the greatest metastatic potential and poorest clinical outcome was significant higher than that in SLHCC and SHCC with relatively better clinical outcome [2, 24] (Figure 1D1). But the median of JARID2 expression in SHCC was similar to that in SLHCC (Figure 1D1). JARID2 was significantly higher in tumors with microvascular invasion (MVI) than tumors without MVI (Figure 1D2). Patients with metastasis and/or recurrences of HCC also exhibited higher JARID2 mRNA expression than who without metastasis and/or recurrences (Figure 1D3). To confirm the PCR and western blot results, immunohistochemical staining was performed to detect the JARID2 in paraffin-embedded paired HCC samples from training cohort and validation cohort (Supplementary Figure 1 and Supplementary Table 1). The results showed JARID2 protein was primarily localized to the nucleus in HCC (Figure 1E1, Figure 2A) and was highly expressed in 59.4% and 57.6% (69/116; 38/66; Table 1) of HCC tissues, as compared with 12.9% and 3.0% (15/116; 2/66; Supplementary Table 2) in corresponding ANLTs. Its expression was also found to be significantly upregulated in HCC tissues and correlated with HCC subtypes in both training cohort (Figure 1E2) and validation cohort (Figure 1E3). Above of all, these data show that JARID2 is overexpressed in HCC and may contribute to HCC metastasis.

**Table 1:** Correlations between JARID2 expression in HCC tissues and clinicopathologic variables of HCC patients in training and validation cohort

Clinicopathologic variable	Training cohort				Validation Cohort			
	No.	JARID2 expression levels			JARID2 expression level			
		Low	High	*P* value	No.	Low	High	*P* value
**Gender**								
Female	13	5	8	0.874	16	6	10	0.413
Male	103	42	61		50	22	28	
**Age (years)**								
≤ 60	93	39	54	0.805	56	24	32	0.295
> 60	23	8	15		10	4	6	
**AFP(ng/mL)**								
≤ 20	56	24	32	0.620	23	10	13	0.899
> 20	60	23	37		43	18	25	
**HBsAg**								
Negative	29	12	17	0.913	18	6	12	0.182
Positive	87	35	52		48	22	26	
**Liver cirrhosis**								
Absence	31	14	17	0.538	29	13	16	0.725
Presence	85	33	52		37	15	22	
**Child-Pugh classification**								
A	102	41	61	0.849	59	24	35	0.668
B	14	6	8		7	4	3	
**Tumor number**								
Solitary	56	31	25	**0.002**	32	21	11	**< 0.001**
Multiple	60	16	44		34	7	27	
**Tumor size**								
≤ 5 cm	39	13	26	0.262	17	8	9	0.654
> 5 cm	77	34	43		49	20	29	
**Capsular formation**								
Presence	52	26	26	0.061	36	13	23	0.672
Absence	64	21	43		30	15	15	
**Microvascular invasion**								
Absence	68	36	32	**0.001**	41	23	18	**0.004**
Presence	48	11	37		25	5	20	
**Edmondson-Steiner grade**								
Low grade (I and II)	36	22	14	**0.004**	30	19	11	**0.002**
High grade (III and IV)	80	25	55		36	9	27	
**HCC subtype**				**0.007**^#^				**0.001**^#^
SHCC	24	14	10	0.698^##^	14	9	5	0.890^##^
SLHCC	32	17	15	0.006^*^	18	12	6	0.004^*^
NHCC	60	16	44	0.012^**^	34	7	27	0.001^**^
**TNM Stage**								
I	51	26	25	**0.042**	27	17	10	**0.005**
II–III	65	21	44		39	11	28	
**BCLC Stage**								
0–A	49	25	24	**0.049**	21	14	7	**0.006**
B–C	67	22	45		45	14	31	

**Abbreviations: AFP**, alpha-fetoprotein; **TNM**, tumor node metastasis; **BCLC**, Barcelona Clinic Liver Cancer; **SHCC**, small hepatocellular carcinoma; **SLHCC**, solitary large hepatocellular carcinoma; **NHCC**, nodular hepatocellular carcinoma;

# SHCC *vs*. SLHCC *vs*. NHCC;

## SHCC *vs*. SLHCC;

* SHCC *vs*. NHCC;

** SLHCC *vs*. NHCC.

### High JARID2 expression associates with the clinicopathologic features for HCC

Next, we sought to explore the association of JARID2 expression with the clinicopathologic features for HCC in two independent cohorts-training and validation cohort from two research centers (Supplementary Figure 1 and Supplementary Table 1). In training cohort, high JARID2 expression in HCC tissues positively correlated with tumor number (*P* = 0.002), microvascular invasion (*P* = 0.001), Edmondson-Steiner grade (*P* = 0.004), HCC subtype (*P* = 0.007), TNM stage (*P* = 0.042) and BCLC stage (*P* = 0.049) (Table 1). However, high JARID2 expression in HCC tissues did not correlate with gender, age, HBV infection, AFP, presence of cirrhosis, size of the tumor and presence of encapsulation (Table 1). The similar results were further validated in the validation cohort (Table 1). Data showed that high JARID2 expression in HCC tissues also positively correlated with tumor number (*P* < 0.001), microvascular invasion (*P* = 0.004), Edmondson-Steiner grade (*P* = 0.002), HCC subtype (*P* = 0.001), TNM stage (*P* = 0.005) and BCLC stage (*P* = 0.006) (Table 1). We also did not detect correlations between high JARID2 expression in HCC tissues and gender, age, HBV infection, AFP, presence of cirrhosis, size of the tumor and presence of encapsulation (Table 1). Notably, data showed no significant association JARID2 expression in ANLTs with the clinicopathologic features for HCC in training and validation cohort (Supplementary Table 2).

### High expression level of JARID2 correlates with poor prognosis for HCC patients

To further evaluate the prognostic potential of JARID2 expression in HCC tissues, a univariate analysis was first performed followed by the multivariate Cox proportional hazards analysis. In training cohort (Supplementary Figure 1 and Supplementary Table 1), the results showed that, in addition to tumor numbers, capsular formation, microvascular invasion, TNM stage and BCLC stage, high JARID2 expression in HCC tissues was also found to be a significant independent prognosis factor for disease-free survival (DFS) (HR 1.641; 95% CI: 1.294 to 3.102; *P* = 0.017; Table 2) and overall survival (OS) (HR 1.873; 95% CI: 1.108 to 3.845; *P* = 0.041; Table 3). Analyzed by the Kaplan-Meier method with log-rank test, high tumor JARID2 expression were found to be associated with lower DFS (1-, 3- and 5-year DFS: 63.2%, 35.5%, 17.9% *vs.* 87.2%, 68.1%, 37.5%, *P* = 0.001; Figure 2B1), lower OS (1-, 3- and 5-year OS: 78.1%, 63.0%, 25.1% *vs.* 89.4%, 80.9%, 48.2%, *P* = 0.002; Figure 2B2) and a significantly higher early recurrence rate (recurrence within 2 years: 47.8% *vs.* 21.3%, *P* = 0.003; Figure 2B3) than patients with low JARID2 expression. In line with this result, NHCC had lower DFS (1-, 3- and 5-year DFS (NHCC *vs.* SHCC *vs.* SLHCC): 35.1%, 83.3%, 70.2% *vs.* 32.0%, 81.3%, 62.5%, *vs.* 17.1%, 63.1%, 32.4%, *P* = 0.001; Figure 2C1) and lower OS (1-, 3- and 5-year OS (NHCC *vs.* SHCC *vs.* SLHCC): 48.0%, 91.7%, 82.9% *vs.* 42.3%, 87.5%, 78.1% *vs.* 26.6%, 74.8%, 47.6%, *P* = 0.002; Figure 2C2) rates than SHCC and SLHCC.

**Table 2:** The cox proportional hazard regression analyses for disease-free survival in training and validation cohort

Variables	Training Cohort					Validation Cohort				
	No.	Univariable Analysis		Multivariable Analysis		No.	Univariable Analysis		Multivariable Analysis	
		HR (95% CI)	*P* Value	HR (95% CI)	*P* Value		HR (95% CI)	*P* Value	HR (95% CI)	*P* Value
**Gender**										
Female	13	Reference				16	Reference			
Male	103	1.034 (0.526–1. 745)	0.514		NA	50	1.718 (0.704–3.109)	0.331		NA
**Age (years)**										
≤ 60	93	Reference				56	Reference			
> 60	23	1.324 (0.814–2.183)	0.241		NA	10	1.207 (0.572–2.137)	0.172		NA
**AFP(ng/mL)**										
≤ 20	56	Reference				23	Reference			
> 20	60	1.018 (0.406–1.803)	0.433		NA	43	1.216 (0.835–2.238)	0.164		NA
**HBsAg**										
Negative	29	Reference				18	Reference			
Positive	87	1.176 (0.739–2.833)	0.397		NA	48	1.675 (0.802–2.783)	0.418		NA
**Liver cirrhosis**										
Absence	31	Reference				29	Reference			
Presence	85	1.199 (0.733–1.961)	0.312		NA	37	1.023 (0.562–2.093)	0.732		NA
**Child-Pugh classification**										
A	102	Reference				59	Reference			
B	14	1.342 (0.763–2.360)	0.230		NA	7	1.034 (0.732–1.901)	0.290		NA
**Tumor number**										
Solitary	56	Reference		Reference		32	Reference		Reference	
Multiple	60	2.175 (1.532–4.782)	**0.007**	1.773 (1.349–3.082)	**0.029**	34	2.179 (1.723–3.835)	**0.016**	1.832 (1.214–2.907)	**0.031**
**Tumor size**										
≤ 5 cm	39	Reference				17	Reference			
> 5 cm	77	1.316 (0.782–1.894)	0.342		NA	49	1.617 (0.883–2.308)	0.239		NA
**Capsular formation**										
Presence	52	Reference		Reference		36	Reference		Reference	
Absence	64	3.085 (1.546–5.336)	**0.012**	1.432 (1.083–3.348)	**0.035**	30	2.285 (1.517–3.934)	**0.008**	1.864 (1.356–2.973)	**0.027**
**Microvascular invasion**										
Absence	68	Reference		Reference		41	Reference		Reference	
Presence	48	2.483 (1.758–4.872)	**0.009**	2.014 (1.389–3.735)	**0.021**	25	2.782 (1.948–4.083)	**0.003**	1.918 (1.534–3.235)	**0.012**
**Edmondson-Steiner grade**										
Low grade (I and II)	36	Reference		Reference		30	Reference			
High grade (III and IV)	80	1.521 (1.034–2.416)	**0.045**	1.007 (0.634–1.629)	0.102	36	1.216 (0.835–2.238)	0.403		NA
**TNM Stage**										
I	51	Reference		Reference		27	Reference		Reference	
II – III	65	3.634 (2.043–7.346)	**0.003**	2.206 (1.804–4.827)	**0.013**	39	2.209 (1.284–3.923)	**0.010**	1.873 (1.398–3.041)	**0.044**
**BCLC Stage**										
0–A	49	Reference		Reference		21	Reference		Reference	
B–C	67	2.563 (1.843–5.054)	**0.008**	1.872 (1.083–3.823)	**0.032**	45	2.386 (1.732–4.939)	**0.007**	2.042 (1.452–3.821)	**0.039**
**Tumor JARID2 expression**										
Low	47	Reference		Reference		28	Reference		Reference	
High	69	3.087 (1.794–4.205)	**0.002**	1.641 (1.294–3.102)	**0.017**	38	3.934 (2.632–6.931)	**< 0.001**	2.463 (1.836–3.352)	**0.033**

**Abbreviations: AFP**, alpha-fetoprotein; **TNM**, tumor node metastasis; **BCLC**, Barcelona Clinic Liver Cancer; **Tumor JARID2 expression**, JARID2 expression in HCC tissues.

**Table 3:** The cox proportional hazard regression analyses for overall survival in training and validation cohort

Variables	Training Cohort					Validation Cohort				
	No.	Univariable Analysis		Multivariable Analysis		No.	Univariable Analysis		Multivariable Analysis	
		HR (95% CI)	*P* Value	HR (95% CI)	*P* Value		HR (95% CI)	*P* Value	HR (95% CI)	*P* Value
**Gender**										
Female	13	Reference				16	Reference			
Male	103	1.076 (0.598–1.932)	0.539		NA	50	1.072 (0.681–1.684)	0.207		NA
**Age (years)**										
≤ 60	93	Reference				56	Reference			
> 60	23	1.285 (0.826–2.332)	0.276		NA	10	1.238 (0.645–2.662)	0.311		NA
**AFP(ng/mL)**										
≤ 20	56	Reference				23	Reference			
> 20	60	1.127 (0.906–1.854)	0.437		NA	43	1.104 (0.804–1.623)	0.412		NA
**HBsAg**										
Negative	29	Reference				18	Reference			
Positive	87	1.174 (0.768–2.159)	0.362		NA	48	1.658 (0.785–2.631)	0.396		NA
**Liver cirrhosis**										
Absence	31	Reference				29	Reference			
Presence	85	1.332 (0.680–2.609)	0.218		NA	37	1.206 (0.660–2.153)	0.254		NA
**Child-Pugh classification**										
A	102	Reference				59	Reference			
B	14	1.439 (0.724–2.860)	0.147		NA	7	1.134 (0.941–2.034)	0.126		NA
**Tumor number**										
Solitary	56	Reference		Reference		32	Reference		Reference	
Multiple	60	3.972 (1.433–6.587)	**< 0.001**	1.795 (1.187–2.753)	**0.047**	34	3.054 (1.625–5.154)	**0.001**	1.837 (1.351–2.985)	**0.021**
**Tumor size**										
≤ 5 cm	39	Reference				17	Reference			
> 5 cm	77	1.306 (0.969–1.846)	0.158		NA	49	1.402 (0.743–2.851)	0.343		NA
**Capsular formation**										
Presence	52	Reference		Reference		36	Reference		Reference	
Absence	64	3.834 (1.972–7.624)	**0.014**	2.036 (1.288–4.732)	**0.029**	30	1.506 (1.041–2.198)	**0.040**	1.145 (0.837–1.517)	0.109
**Microvascular invasion**										
Absence	68	Reference		Reference		41	Reference		Reference	
Presence	48	3.459 (1.645–4.252)	**0.002**	2.678 (1.437–3.107)	**0.012**	25	2.755 (1.952–4.245)	**0.009**	2.028 (1.556–3.551)	**0.013**
**Edmondson-Steiner grade**										
Low grade (I and II)	36	Reference		Reference		30	Reference			
High grade (III and IV)	80	1.381 (1.012–2.783)	**0.037**	1.064 (0.804–1.801)	0.094	36	1.542 (0.955–2.275)	0.302		NA
**TNM Stage**										
I	51	Reference		Reference		27	Reference		Reference	
II–III	65	2.728 (1.579–5.668)	**0.017**	1.625 (1.256–3.254)	**0.039**	39	2.661 (1.108–6.356)	**0.012**	1.814 (1.581–3.861)	**0.047**
**BCLC Stage**										
0–A	49	Reference		Reference		21	Reference		Reference	
B–C	67	3.137 (1.791–6.138)	**0.001**	2.506 (1.231–5.097)	**0.026**	45	2.796 (1.985–4.643)	**0.015**	1.735 (1.352–3.192)	**0.041**
**Tumor JARID2 expression**										
Low	47	Reference		Reference		28	Reference		Reference	
High	69	2.956 (1.351–5.014)	**0.005**	1.873 (1.108–3.845)	**0.041**	38	2.963 (1.704–5.162)	**0.018**	2.241 (1.568–3.811)	**0.031**

**Abbreviations: AFP**, alpha-fetoprotein; **TNM**, tumor node metastasis; **BCLC**, Barcelona Clinic Liver Cancer; **Tumor JARID2 expression**, JARID2 expression in HCC tissues.

To estimate the accuracy of JARID2 expression in HCC tissues to predict prognosis, data from the validation cohort (Supplementary Figure 1 and and Supplementary Table 1) were further calculated. In the validation cohort, high JARID2 expression in HCC tissues was still found to be an independent risk factor for DFS (HR 2.463; 95% CI: 1.836 to 3.352; *P* = 0.033; Table 2) and OS (HR 2.241; 95% CI: 1.568 to 3.811; *P* = 0.031; Table 3). HCC patients with high JARID2 expression had lower DFS (1-, 3- and 5-year DFS: 73.7%, 39.5%, 11.2% *vs.*85.7%, 67.9%, 50.0%, *P* = 0.002; Figure 2D1), lower OS (1-, 3- and 5-year OS: 78.9%, 55.3%, 24.9% *vs.* 89.3%, 75.0%, 57.1%, *P* = 0.001; Figure 2D2) and a significantly higher early recurrence rate (recurrence within 2 years: 50% *vs.* 17.9%, *P* = 0.010; Figure 2D3) than who with low JARID2 expression. In line with this result, NHCC had lower DFS (1-, 3- and 5-year DFS (NHCC *vs.* SHCC *vs.* SLHCC): 42.9%, 85.7%, 64.3% *vs.* 38.9%, 83.3%, 61.1% *vs.* 14.7%, 73.5%, 41.2%, *P* = 0.044; Figure 2E1) and OS (1-, 3- and 5-year OS (NHCC *vs.* SHCC *vs.* SLHCC): 57.1%, 85.7%, 71.4% *vs.* 50.0%, 83.3%, 66.7% *vs.* 27.9%, 82.4%, 55.9%, *P* = 0.024; Figure 2E2) rates than SHCC and SLHCC. Taken together, these data suggest that increased JARID2 expression in HCC tissues predicts poor prognosis in HCC patients and may contribute to HCC metastasis and malignant progression.

### JARID2 promotes invasion and metastasis of HCC Cells *in vitro* and *in vivo*

**Figure 3:**
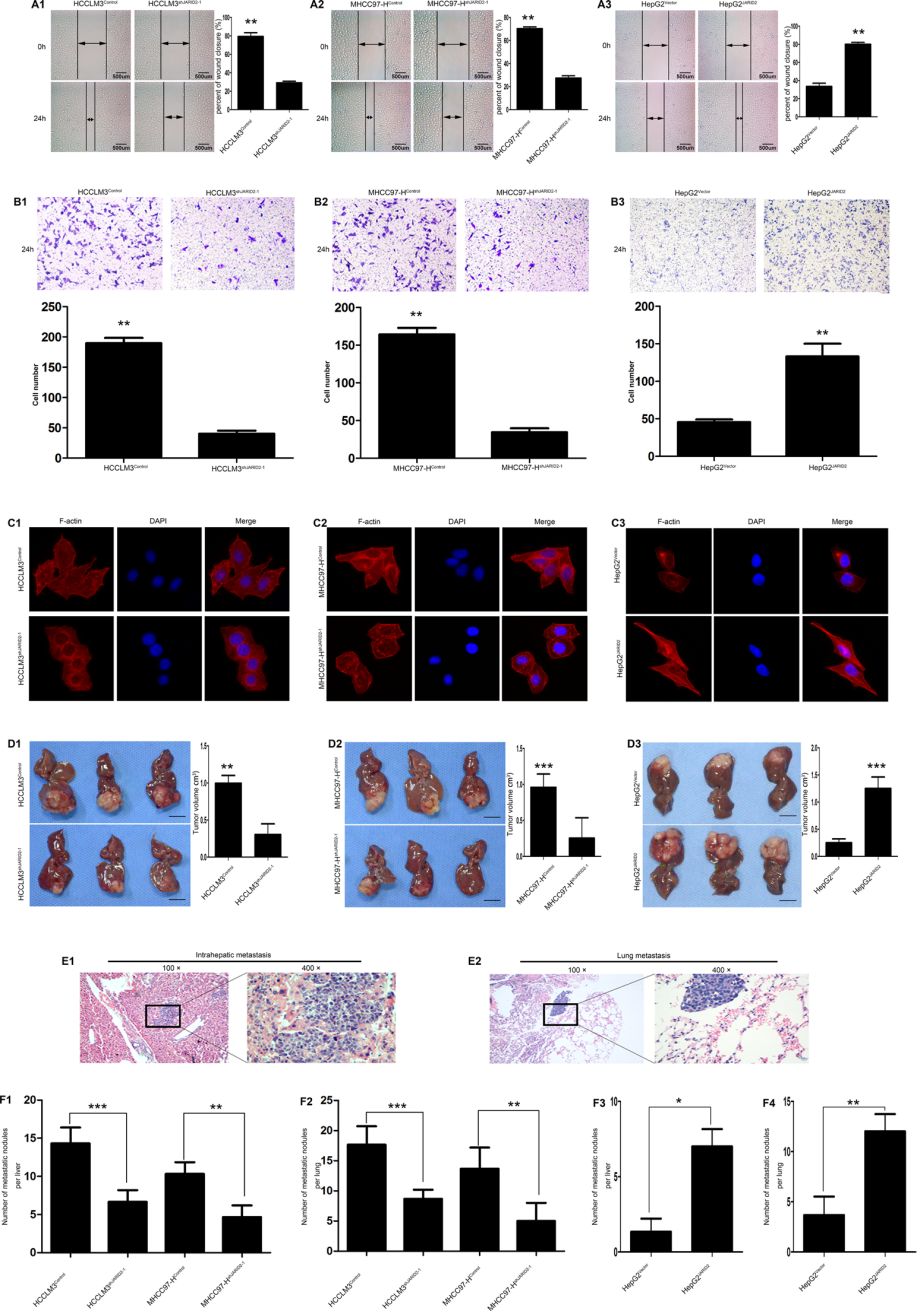
JARID2 significantly promotes invasion and metastasis of HCC cells *in vitro* and *in vivo* (**A**) Knockdown of JARID2 expression inhibited HCCLM3 (**A1**) and MHCC97-H (**A2**) cells migration, whereas overexpressed JARID2 in HepG2 cells promoted cell migration (**A3**) in wound-healing assays. (**B**) The transwell assays showed that knockdown of JARID2 expression inhibited HCCLM3 (**B1**) and MHCC97-H (**B2**) cells invasion, but JARID2 overexpression enhanced HepG2 cells (**B3**) invasion. (**C**) Immunofluorescence assays of cytoskeleton of HCCLM3^control^ and HCCLM3^shJARID2-1^ (**C1**), MHCC97-H^control^ and MHCC97-H^shJARID2-1^ (**C2**), HepG2^Vector^ and HepG2^JARID2^ cells (**C3**). F-actin filaments were visualized in cells using rhodamine-phalloidin. (**D**) The HCC metastatic mouse model was constructed by using HCCLM3 and MHCC97-H cells transfected with shJARID2-1 or control vector, and HepG2 cells transfected with JARID2 or control vector as described in the Materials and Methods. The size of liver tumors in each group was calculated and compared in HCCLM3 (**D1**), MHCC97-H (**D2**) and HepG2 (**D3**) cells. Scale bar, 1 cm. (**E**) Representative pictures for intrahepatic (**E1**) and lung metastasis (**E2**). (**F**) The number of metastatic nodules per liver or lung was calculated and compared between HCCLM3^control^ and HCCLM3^shJARID2-1^ (**F1**), MHCC97-H^control^ and MHCC97-H^shJARID2-1^ (**F2**), HepG2^Vector^ and HepG2^JARID2^ cells group (**F3**, **F4**). *P < 0.05; **P < 0.01; ***P < 0.001.

The difference of JARID2 expression was examined in a normal liver cell line (L02 cells) and five HCC cell lines with various metastatic potential by qRT-PCR (Supplementary Figure 2A1), RT-PCR (Supplementary Figure 2A2) and western blot (Supplementary Figure 2B). The results showed that HCCLM3 cells possessing the highest capability for metastasis had the highest JARID2 expression among the five HCC cell lines. According to the expression level of JARID2 in HCC cell lines and biological characteristics of HCC cell lines, [25, 26] we overexpressed JARID2 in HepG2 cells (Supplementary Figure 2C) and stably knocked down JARID2 in HCCLM3 (Supplementary Figure 2D), MHCC97-H cells (Supplementary Figure 2E). Wound healing and transwell assays were used to analyze the effect of JARID2 on migration and invasion ability of HCC cells. The wound healing assays showed that down-regulated expression of JARID2 in HCCLM3 cells was associated with significantly slow wound closure (Figure 3A1; Supplementary Figure 3A1). Transwell assays showed that, comparing with HCCLM3^control^ cells, HCCLM3^shJARID2^ cells exhibit a significant reduction in the number of invaded cells (Figure 3B1; Supplementary Figure 3B1). Similarly, reduced JARID2 expression in MHCC97-H cells also resulted in significantly slow wound closure (Figure 3A2; Supplementary Figure 3A2) and a significant reduction in the number of invaded cells (Figure 3B2; Supplementary Figure 3B2). Conversely, JARID2 overexpression significantly enhanced motility of HepG2 cells by wound healing assay (Figure 3A3) and increased invasive ability of HepG2 cells by transwell assays (Figure 3B3). Immunofluorescence staining of F-actin was used to analyze cytoskeleton. The results showed that the stress fiber-like structures disappeared in HCCLM3^shJARID2^ cells compared to HCCLM3^control^ cells (Figure 3C1; Supplementary Figure 3C1). The similar results were shown in MHCC97-H cells (Figure 3C2; Supplementary Figure 3C2). Whereas overexpression of JARID2 promoted the reorganization of F-actin to form cytoskeleton in HepG2 cells (Figure 3C3). Analysis of colony formation or cell proliferation showed an effect of JARID2 on promoting cell proliferation in HCCLM3 (Supplementary Figure 4A, 4B; Supplementary Figure 5A, 5B), MHCC97-H cells (Supplementary Figure 4C, 4D; Supplementary Figure 5C, 5D) and HepG2 (Supplementary Figure 4E, 4F). These findings implicate that JARID2 functions as an oncogene and is involved in HCC cell invasion.

Next, the effect of JARID2 on HCC invasion and metastasis was analyzed by *in vivo* metastatic assays. The mouse xenograft orthotopic liver cancer model assays showed that the growth of tumors (in terms of mean tumor volume) originally formed from HCCLM3^shJARID2^ cells were significantly smaller than that from HCCLM3^Control^ cells (Figure 3D1), and the mean tumor volume of the MHCC97-H^shJARID2^ group was also significantly smaller than that of the MHCC97-H *Control* group (Figure 3D2), but the mean tumor volume of the HepG2^JARID2^ group was also significantly larger than that of the HepG2^Control^ group (Figure 3D3). Consistently, the number of metastatic nodules in the liver (Figure 3E1) or lung (Figure 3E2) in mice implanted with JARID2-knockdown cells was significantly smaller than that mice implanted with cells transfected with the control vector (Figure 3F1, 3F2). However, the number of metastatic nodules in the liver (Supplementary Figure 6A) or lung (Supplementary Figure 6B) in mice implanted with JARID2-overexpressed cells was significantly larger than that mice implanted with cells transfected with the control vector (Figure 3F3, 3F4). These data suggest that JARID2 promotes tumor formation and metastasis.

### JARID2 promotes HCC invasion and metastasis by inhibiting PTEN expression through H3K27 trimethylation

**Figure 4:**
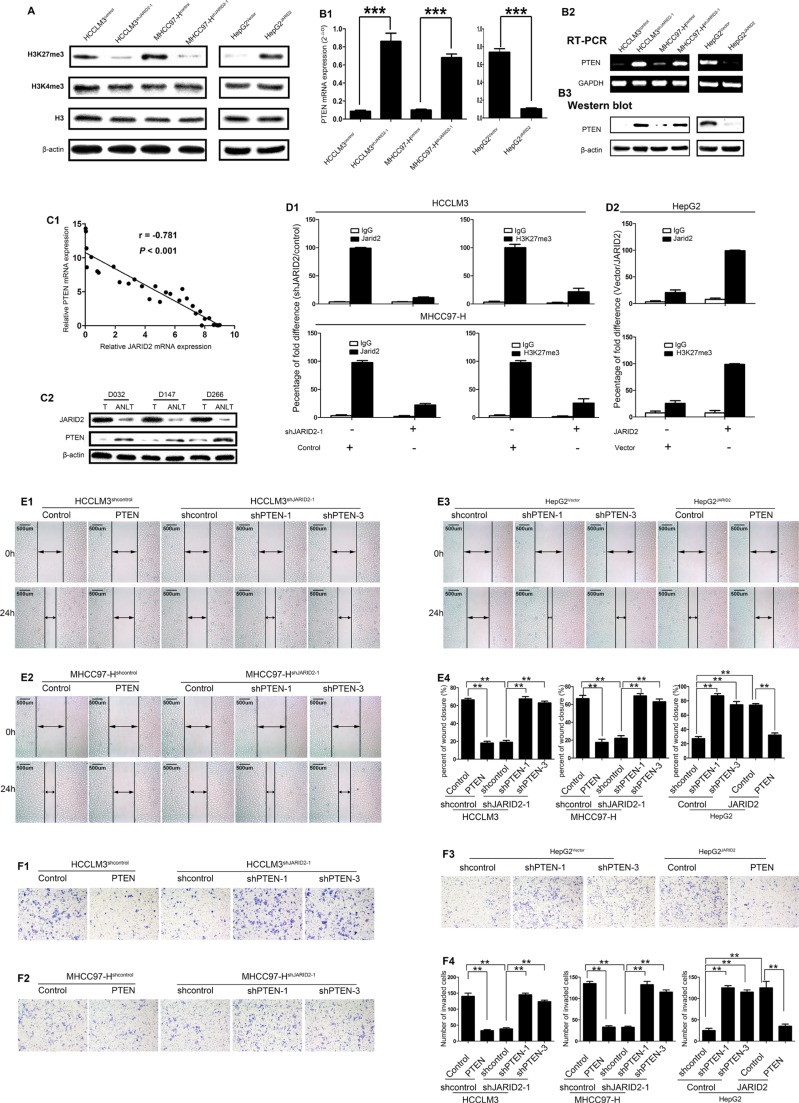
JARID2 Promotes Metastasis of Hepatocellular Carcinoma by inhibiting PTEN Expression through H3K27 Trimethylation (**A**) Western blot analysis showed, compared with control cells, a decrease in H3K27me3 in HCCLM3shJARID2-1 and MHCC97-H^shJARID2-1^ cells, but an increase in H3K27me3 in HepG2^JARID2^ cells. Histones H3 and β-actin were used as loading controls. (**B**) PTEN expression was upregulated in HCC cells with JARID2 knockdown and downregulated in HCC cells with JARID2 overexpression. The expression levels of PTEN was measured in HCCLM3, MHCC97-H cells with JARID2 knockdown, HepG2 cells with JARID2 overexpression and their corresponding control by qPCR (**B1**), semiquantitative RT-PCR (**B2**), western blot (**B3**). (**C**) The expression level of JARID2 exhibited an inverse trend with that of PTEN in HCC samples. The expression of JARID2 and PTEN was detected in 30 paired HCC tissues and ANLTs. (**C1**) The relative level of JARID2 mRNA expression was plotted against the relative level of PTEN mRNA expression. (**C2**) Western blot detected the expression of JARID2 and PTEN protein in the same HCC tissues and ANLTs. (**D**) JARID2 was associated with increased H3K27me3 at PTEN promoter region. Quantitative chromatin immunoprecipitation (qChIP) was used to assess H3K27me3 at PTEN promoters region in HCCLM3, MHCC97-H cells with shJARID2 knockdown, HepG2 cells with JARID2 overexpression and their corresponding control vector. qChIP was performed using the indicated antibodies. Purified rabbit IgG was a negative control. The percentage fold difference of shJARID2/control, Vector/JARID2 was calculated as the difference in percentage of the qChIP in HCCLM3, MHCC97-H cells transfected with shJARID2 relative to cells transfected with control vector, in HepG2 cells transfected with control vector relative to cells transfected with JARID2 vector respectively. Each bar represents the mean ± SEM for triplicate experiments. (E, F) Gain- and loss-off function studied with PTEN expression vector, shPTENs or its control showed PTEN is a critical downstream effector in JARID2-promoted invasion and metastasis in HCC. HCC cells were transfected with shRNAs for PTEN or PTEN expression vector to inhibit or restore the PTEN expression. Wound-healing assays (E) and transwell assays (F) were determined in HCCLM3, MHCC97-H, HepG2 cells. **, *P* < 0.01; ***, *P* < 0.001.

To define the mechanisms by which JARID2 promotes HCC invasion and metastasis, we tried to identify potential targeted genes regulated by JARID2. It was reported that JARID2 physically associated with PRC2 and was functionally involved in trimethylation of histone H3 lysine 27 (H3K27me3) [12, 13]. Likewise, the interaction between JARID2 and PRC2 was further validated by coimmunoprecipitation (co-IP) in HCC cells (Supplementary Figure 7A–7C). To further explore JARID2 is functionally together with PRC2 in HCC, we next investigated whether JARID2 knockdown or overexpression would affect H3K27me3 expression in HCC cells. Western blot showed JARID2 knockdown decreased the level of H3K27me3 expression, whereas JARID2 overexpression increased the level of H3K27me3 expression (Figure 4A). However, H3K4me3 was not affected (Figure 4A).

PRC2 could inhibit the transcription of PTEN, [23] and JARID2 is functionally together with PRC2 in HCC. Notably, PTEN plays a critical role in HCC proliferation, invasion and metastasis, [20] supporting the function of JARID2 in regulating proliferation, invasion and metastasis. These also suggest that JARID2 possibly coordinates with PRC2 to exert functions by inhibiting PTEN expression in HCC. As expected, JARID2 knockdown in HCCLM3 or MHCC97-H cells led to increased expression of PTEN at both the transcriptional level (Figure 4B1, 4B2) and the protein level (Figure 4B3). On the contrary, JARID2 overexpression in HepG2 cells resulted in decreased expression of PTEN at both the transcriptional level (Figure 4B1, 4B2) and the protein level (Figure 4B3). Furthermore, the expression level of JARID2 showed an inverse trend with that of PTEN in HCC samples, as measured by qRT-PCR (Figure 4C1) and western blotting (Figure 4C2). These data support the observation that PTEN are suppressed by JARID2 at the transcriptional level. Notably, quantitative chromatin immunoprecipitation (qChIP) assays showed the inhibition of JARID2 expression led to a strong reduction of JARID2 binding to PTEN promoter region and, consistent with this, to a reduction of H3K27me3 levels at PTEN promoter region (Figure 4D1), whereas JARID2 overexpression led to a dramatic increase of JARID2 binding to PTEN promoter region and, similarly, to an increase of H3K27me3 levels at PTEN promoter region (Figure 4D2). These findings indicate that JARID2 represses the expression of PTEN through increasing H3K27me3 at PTEN promoter region.

To determine whether PTEN is a mediator for JARID2-promoted invasion and metastasis in HCC, a complementary approach of gain- and loss-off function of PTEN was used. PTEN was overexpressed in HCCLM3^shcontrol^ (Supplementary Figure 7D), MHCC97-H^shcontrol^ (Supplementary Figure 7E), HepG2^JARID2^ (Supplementary Figure 7F) with PTEN expression vector and knockdown in HCCLM3^shJARID2^ (Supplementary Figure 7D), MHCC97-H^shJARID2^ cells (Supplementary Figure 7E), HepG2^Vector^, (Supplementary Figure 7F). The overexpression of PTEN in HCCLM3*shcontrol*, MHCC97-H^shcontrol^ inhibited the migration and invasion (Figure 4E1, 4E2, 4E4 and 4F1, 4F2, 4F4). Moreover, downregulation of PTEN expression abolished the effect of JARID2 knockdown on migration and invasion in HCCLM3^shJARID2^ or MHCC97-H^shJARID2^ cells (Figure 4E1, 4E2, 4E4 and 4F1, 4F2, 4F4). In contrast, silence of PTEN expression mimicked the effect of JARID2 on migration and invasion capacity in HepG2^Vector^ cells (Figure 4E3, 4E4 and 4F3, 4F4), while overexpression of PTEN attenuated JARID2-promoted migration and invasion in HepG2^JARID2^ cells (Figure 4E3, 4E4 and 4F3, 4F4). In addition, ectopic expression of PTEN reduced the proliferation and colony formation of HCCLM3^shcontrol^ (Supplementary Figure 8A1, 8A2 and 8D), MHCC97-H^shcontrol^ cells (Supplementary Figure 8B1, 8B2 and 8E) and inhibition of PTEN expression restored the ability of the proliferation and colony formation of HCCLM3^shJARID2^ (Supplementary Figure 8A1, 8A2 and 8D), MHCC97-H^shJARID2^ cells (Supplementary Figure 8B1, 8B2 and 8E), while silence of PTEN expression mimicked the effect of JARID2 on colony formation ability or cell proliferation in HepG2^Vector^ cells and overexpression of PTEN expression abolished the effect of JARID2 on colony formation ability and cell proliferation in HepG2^JARID2^ cell (Supplementary Figure 8C1, 8C2 and 8F). Collectively, these results indicate that JARID2 promotes HCC invasion and metastasis by suppressing PTEN expression via increasing H3K27me3 at its promoter region.

### JARID2 promotes metastasis of HCC via facilitating EMT through activating PTEN/AKT Signaling Pathway

HCCLM3 cells with high metastatic capability exhibit high expression of EMT markers [27]. Interestingly, it was observed that HCCLM3 cells exhibited high JARID2 expression (Supplementary Figure 2) and knockdown of JARID2 resulted in the cell appearance with more cobble-like, epithelial morphology in HCCLM3 (Figure 3C1) and MHCC97-H cells (Figure 3C2), whereas overexpression of JARID2 changed the cell appearance to more spindle-like, fibroblastic morphology in HepG2 cells (Figure 3C3). Thus, these indicated that JARID2 might promote metastasis of HCC via EMT.

**Figure 5:**
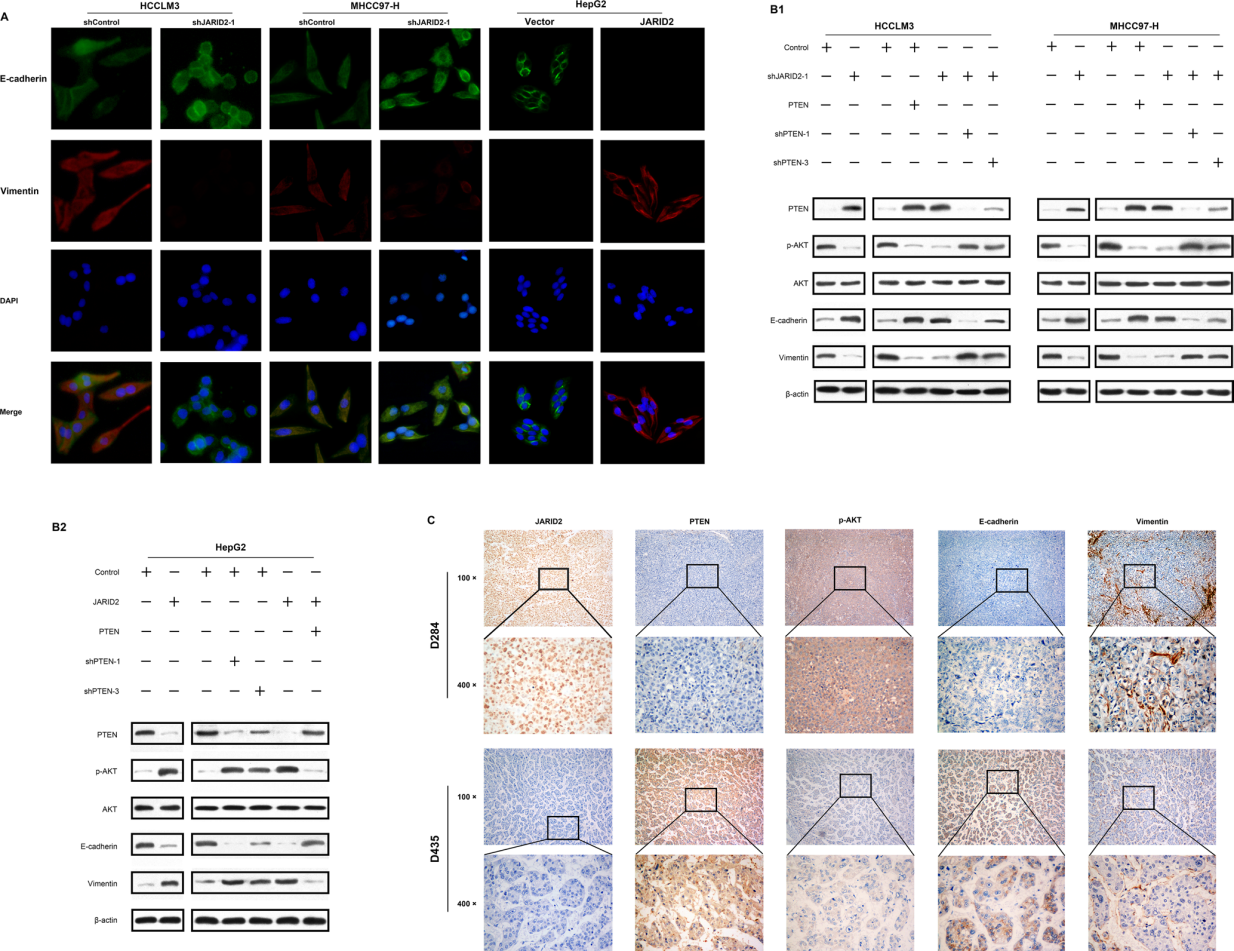
JARID2 promotes epithelial-mesenchymal transition through PTEN/AKT signaling (**A**) Immunofluorescence (**IF**) showed the relative expression of E-cadherin (green), vimentin (red), and 4′,6-diamidino-2-phenylindole (DAPI; blue) in HCC cells with JARID2 knockdown, JARID2 overexpression and their corresponding control vector. (**B**) Western blot analysis of PTEN, AKT, p-AKT, E-cadherin, and vimentin expression in HCC cells with JARID2 knockdown, JARID2 overexpression and their corresponding control vector, and these HCC cells with PTEN silence, ectopic expression and their corresponding control. (**C**) Immunohistochemistry (IHC) analysis of JARID2, PTEN, p-AKT, E-cadherin, and vimentin expression in human HCC tissues. Representative images were from a sample (D284) with high JARID2 expression and a sample (D435) with low JARID2 expression. Original magnification: up, 100×; down, 400×, respectively.

To further confirm whether JARID2 promotes EMT, immunofluorescence (IF) analyses showed knockdown of JARID2 in HCCLM3 and MHCC97-H significantly reduced the expression level of mesenchymal marker vimentin, but significantly increased the expression level of epithelial marker E-cadherin (Figure 5A). Conversely, overexpression of JARID2 in HepG2 cells significantly increased the expression level of mesenchymal marker vimentin, but dramatically reduced the expression level of epithelial marker E-cadherin (Figure 5A). Similar with IF results, western blotting also showed that JARID2 knockdown in HCCLM3 and MHCC97-H decreased the expression of vimentin and increased the expression of E-cadherin (Figure 5B1). However, JARID2 overexpression in HepG2 increased the expression of vimentin and decreased the expression of E-cadherin (Figure 5B2). Moreover, the changes of these EMT markers and JARID2 in protein expression were also observed in transplanted orthotropic tumors of mice (Supplementary Figure 9A and 9B). Furthermore, IHC showed high JARID2 expression was co-location with low E-cadherin and high vimentin expression in HCC samples (Figure 5C). A similar correlation of the expression of E-cadherin, vimentin with the expression of JARID2 was also observed in the HCC tissues (Supplementary Table 3). These results suggest that JARID2-promoted HCC invasion and metastasis is associated with EMT.

Previous studies have demonstrated that PTEN negatively regulated AKT signaling pathway, which is important for EMT [28, 29]. Western blotting was performed to examine whether JARID2 suppressed PTEN expression and subsequently activated AKT signaling to facilitate EMT. Our data showed that inhibition of JARID2 reduced phosphorylation level of AKT (Figure 5B1), and was associated with decreased vimentin expression and increased E-cadherin expression (Figure 5B1). In contrast, overexpression of JARID2 up-regulated phosphorylation level of AKT (Figure 5B2), and was associated with increased vimentin expression and decreased E-cadherin expression (Figure 5B2). Furthermore, western blotting showed suppression of PTEN in JARID2-knockdown cells rescued AKT activities of HCC cells (Figure 5B1), whereas up-regulation of PTEN abolished the AKT activities induced by JARID2 ectopic expression (Figure 5B2). Moreover, IHC showed that HCC tissues with higher JARID2 expression, weaker PTEN expression exhibited stronger staining of p-AKT (Figure 5C). The similar results were also observed in transplanted orthotropic tumors of mice (Supplementary Figure 9A abd 9B). The correlation analysis revealed JARID2 expression was negatively correlative with PTEN expression, but positively correlative with p-AKT level in HCC sample (Supplementary Table 3). Furthermore, PTEN expressions reversely correlated with p-AKT level, vimentin expression and positively with E-cadherin in HCC sample (Supplementary Table 4). Taken together, these data indicate that JARID2 promotes EMT by suppressing PTEN expression and subsequently activating AKT signaling.

## DISCUSSION

Recurrence and metastasis remains the main reason for poor prognosis of HCC after curative liver resection, [3, 30] and the molecular mechanisms underlying HCC recurrence and metastasis have not been thoroughly understood. Although JARID2 was studied in the malignancies such as rhabdomyosarcoma and leukemia, [15, 17] it remains unclear whether and how JARID2 contributes to HCC progression. In this study, we found that JARID2 was significantly upregulated in human HCC tissues compared with ANLTs. qRT-PCR showed that JARID2 mRNA was significantly higher in primary tumors with microvascular invasion (MVI) than those without MVI. Patients with metastasis and/or recurrence had higher JARID2 mRNA expression than those without metastasis and/or recurrence. Interestingly, NHCC with the greatest metastatic potential and poorest clinical outcome had the highest JARID2 expression in comparison to SLHCC and SHCC, which had relatively better clinical outcome based on our previous clinical studies [2, 24]. The differential expression of JARID2 among three subtypes is an another important evidence to the diversity of molecular biological characters and consolidate our evidence that differentially expressed gene profiles between SLHCC and NHCC [31]. IHC also demonstrated that high JARID2 protein expression was significantly correlated with multiple tumor numbers, MVI, high Edmondson-Steiner grade, advanced TNM stage and BCLC stage. The presence of MVI in surgical specimens is known to be an important cause of HCC recurrence after resection, and generally have a poor prognosis [32]. Kaplan-Meier analysis showed HCC patients with high JARID2 expression had worse clinical outcomes than those with low JARID2 expression. A multivariate analysis revealed that high JARID2 expression in HCC tissues was also found to be a significant and independent prognosis factor for DFS and OS. Moreover, high JARID2 expression in HCC tissues was tested to be a good predictor for HCC early recurrence (within 2 years), which is typically caused by dissemination of metastatic HCC cells [33]. This indicates that we may identify the subgroup of HCC patients who are at high risk of developing metastatic disease in advance according to JARID2 expression in HCC tissues. The predictive value of prognosis based on JARID2 expression level further validated in validation cohort, indicating the anticipated availability as a novel molecular biomarker. These data also suggest that JARID2 contributes to HCC invasion and metastasis.

A recent study reported that JARID2 promoted invasion of A549 lung cancer cell line and HT29 colon cancer cell line by the *in vitro* assays [17]. Here we showed that JARID2 overexpression was associated with metastatic potential of HCC cells. Downregulation of endogenous JARID2 expression in HCC cells suppressed cell migration and invasion, while ectopic JARID2 expression significantly promoted cell migration and invasion *in vitro*. Moreover, the *in vivo* metastatic assays showed that downregulation of JARID2 expression significantly decreased intrahepatic and lung metastasis, whereas JARID2 overexpression obviously increased intrahepatic and lung metastasis *in vivo*. Above of all, our data comprehensively shows that JARID2 promotes HCC invasion and metastasis.

The underlying mechanism of JARID2 in promoting HCC invasion and metastasis need to be further elucidated. In this study, we showed that PTEN was the key downstream effector of JARID2. PTEN is often down-regulated in cancers and is associated with advanced stages of cancers or metastasis, [21, 34] including HCC.[22, 35] Our data uncovered that JARID2 was associated with increasing H3K27me3, not H3K4me3, at the PTEN promoter region in HCC, and this resulted in down-regulation of PTEN expression, which consequently promotes HCC cell invasion and metastasis. By gain- and loss-off function assays, we also exhibited that overexpression of PTEN in high JARID2 expression HCC cells attenuated JARID2-promoted invasion and metastasis, while depletion of PTEN in JARID2-knockdown HCC cells could mimick the roles of JARID2. Thus, these results show that JARID2 transcriptionally represses PTEN expression through increasing H3K27me3 at the gene promoter region, and resulting in increase in HCC invasion and metastasis.

An increase in migration and invasion ability is an important character of EMT, which is essential for tumor cells to disseminate to adjacent or distant tissues, and finally causes metastasis-related recurrence [36]. It has been shown that PTEN plays a critical role in the EMT by negative activation of downstream signaling AKT [11, 22]. In HCC, activation of AKT signaling is frequently observed due to loss of PTEN, [20, 35] which triggers EMT program and endows cancer cells with enhanced metastatic potential [28, 29]. Therefore, it is not difficult to understand that JARID2-mediated suppression of PTEN can activate downstream AKT signaling, and enhance cancer cell invasion ability by inducing EMT in HCC cells. Moreover, this study also proved that JARID2 downregulated PTEN expression, which increased the activated phosphorylation of AKT, consequently enhanced vimentin expression and decreased E-cadherin expression in HCC cells. Notably, PTEN knockdown or overexpression abolished the effects of JARID2 knockdown or overexpression on EMT. Further correlation analysis in HCC samples by IHC showed JARID2 expression was positively associated with p-AKT, vimentin and negatively associated with PTEN, E-cadherin. Furthermore, HCC tissues with low PTEN expression, exhibited higher p-AKT level, higher vimentin expression, but lower E-cadherin expression. All together, these observations support that JARID2 promotes EMT-induced metastasis by suppressing PTEN and activating AKT signaling.

In conclusion, our study shows that JARID2 is significantly upregulated in HCC and high JARID2 expression is associated with poor prognosis, and EMT phenotype in HCC tissue and HCC cell lines. JARID2 promotes HCC invasion and metastasis by facilitating EMT through PTEN/AKT signal pathway. Our data suggest JARID2 functions as a potential oncogene, supporting the pursuit of JARID2 as a prospective therapeutic target for HCC.

## MATERIALS AND METHODS

### HCC Samples

The study was approved by the Ethics Committee of Xiangya School of Medicine, CSU. From January 2009 to December 2010, 30 pairs of frozen fresh tumor liver tissues and corresponding ANLTs were collected after radical surgical resection at Department of surgery, Xiangya Hospital of Central South University. These tissues were used to detect the mRNA and protein expression of JARID2 and PTEN. In addition, normal liver tissues were obtained from 5 patients with hepatic hemangioma for hepatic resection. Another two independent cohorts of subjects including training cohort (*n* = 116) and validation cohort (*n* = 66) from 2 different centers were used for prognostic study according to REMARK guidelines for reporting prognostic biomarkers in cancer [37]. In training cohort, paraffin-embedded paired HCC samples (including HCC tissues and ANLTs) were obtained from HCC patients undergoing radical surgical resection without any preoperative treatment at Department of Surgery, the Xiangya Hospital of Central South University (CSU) from January 2005 to December 2008. In validation cohort, paraffin-embedded paired HCC samples (including HCC tissues and ANLTs) were obtained from HCC patients who underwent radical surgical resection without any preoperative treatment at Department of Abdominal Surgical Oncology, Affiliated Cancer Hospital of Xiangya School of Medicine of CSU from January 2003 to December 2008. All samples randomly selected were histopathologically diagnosed and had completed clinicopathologic and follow-up data. Clinicopathologic characteristics of the patients in training cohort and validation cohort used for prognostic study were shown in Supplementary Table 1 and Supplementary Figure 1. HCC samples were divided into three subgroups as we previously defined: [2, 24] small hepatocellular carcinoma (SHCC, the diameter of HCC ≤ 5 cm), solitary large hepatocellular carcinoma (SLHCC, only one nodule, and diameter > 5 cm, and grows expansively within an intact capsule or pseudocapsule), nodular hepatocellular carcinoma (NHCC, has more than 2 nodules).

### Prognostic study

All HCC patients were regularly followed-up by the same experienced surgical team. The follow-up period was defined as the interval between the date of operation and that of the patient's death or the last follow-up. Deaths from other causes were treated as censored cases. The recurrence and metastasis was surveillance by clinical examination, serial monitoring of alpha-fetoprotein levels and ultrasonography or CT scan or MRI at a 3–4 months' interval. Recurrence and metastasis were diagnosed by clinical examination, serial AFP level, and ultrasonography or computed tomography (CT) scan. Disease-free survival was defined as the length of time after liver resection during which a patient survived without sign of HCC. Data of conventional clinical and pathological variables were also collected for analysis, including age, gender, hepatitis B status, liver cirrhosis, Edmondson-Steiner grade, capsular formation, size of the tumor, number of tumor nodes, microvascular invasion, Child-Pugh classification, TNM stage and BCLC stage. Microvascular invasion was defined as tumor cells forming a thrombus in peritumoral vessels, can only be assessed after careful histological assessment of the whole surgical specimen [38, 39]. The follow-up status and any recurrence were regularly updated in the database for each patient. The study was approved by the Ethics Committee of Xiangya School of Medicine, CSU.

### Cell lines

HepG2 cells were purchased from the American Type Culture Collection (ATCC, Rockville, MD). L02 and SMMC-7721 cells were gifted by the Tumor Institute of Central South University (Changsha, China). MHCC97-L, MHCC97-H and HCCLM3 cells were gifted from the Liver Cancer Institute of Fudan University (Shanghai, China). Short tandem repeat (STR) DNA fingerprinting was used to authenticate all cell lines before experiments. All cell lines were routinely cultured with the high glucose DMEM supplemented with 10% fetal bovine serum, and maintained in 5% CO_2_ humidified incubator at 37°C.

### Vector construction and transfection

The plasmids carrying short hairpin RNAs (shRNAs) for JARID2 knockdown, JARID2 expression vector inserted with JARID2 coding sequences (CDS) for JARID2 overexpression and corresponding controls were purchased from GeneChem Company (Shanghai, China). The sequences of five shRNAs for JARID2 knockdown and its control were shown in Supplementary Table 5. HCCLM3 and MHCC97-H cells were transfected with the shRNA plasmid, and HepG2 cells were transfected with the JARID2 expression plasmid. Cells transfected with control plasmids were used as controls. Transfected cells were selected with 3 μg/mL puromycin. Down-regulated expression or overexpression of JARID2 was confirmed by western blot (Supplementary Figure 2C, 2D, 2E). The inhibitory efficiency of five shRNAs was validated and the JARID2-shRNA-Seq3, JARID2-shRNA-Seq5 (named shJARID2-1, shJARID2-2 respectively in the figures) were adopted for subsequent study (Supplementary Figure 2D and 2E) because of highly effective inhibition of JARID2 expression in HCCLM3 and MHCC97-H cells.

PTEN ectopic expression and knockdown lentivirus as well as their negative control lentivirus were purchased from GeneChem (Shanghai, China). The sequences of shRNA for PTEN were seen in Supplementary Table 5. The PTEN expression vector was constructed by inserting its CDS into the vector under the regulation of a CMV promoter as this might counteract the effects of JARID2-mediated silencing. The lentivirus was transfected into the HCCLM3, MHCC97-H and HepG2 cells with an optimal multiplicity of infection (MOI) of 50 TU/mL. To increase the number of infected cells, the number of lentivirus copies should increase in each HCC cells. Down-regulated expression (at least 80%) or overexpression of PTEN was confirmed by western blot (Supplementary Figure 7D, 7E, 7F). The inhibitory efficiency of three shRNAs for PTEN was validated and the shPTEN-1, shPTEN-3 were adopted for subsequent study.

### Quantitative real-time PCR (qRT-PCR)

Total RNA was extracted from HCC cell lines or fresh frozen tumor specimens by using Trizol reagent (Invitrogen, Carlsbad, CA) according to the manufacturer's instructions. qRT-PCR was performed using the SYBR^*®*^ Green Realtime PCR Master Mix assay kit (Toyobo, Osaka, Japan) according to the manufacturer's instructions. The primers of JARID2 were as follows: forward, 5′-GACACCAAACCCAATCACCAC-3′, reverse, 5′-GTTCAACCTGCCACTGACCTT-3′, GAPDH was used as a control using the following primers: forward, 5′-GCACCGTCAAGGCTGAGAAC-3′, reverse, 5′-TGGTGAAGACGCCAGTGGA -3′. The results were analyzed using the 2^−ΔΔCt^ method as the following formula: ΔΔCt = ΔCt_HCC_ −ΔCt _ANLT,_ ΔCt = Ct_JARID2_ −Ct_GAPDH_.

### Western blot analysis

Total proteins were extracted and separated by sodium dodecyl sulfate-polyacrylamide gel electrophoresis (SDS-PAGE) and then transferred onto PVDF membranes (Millipore, Bedford, MA). The blotted membranes were incubated with the primary antibodies and then an appropriate HRP-conjugated secondary antibody (KPL, Gaithersburg, MD) in order. Band was detected with enhanced chemiluminescence regents (Thermo Scientific, Rockford, IL). Beta-actin protein was also determined by using the specific antibody (Sigma, St Louis, MO) as a loading control. Protein expression were quantified by BandScan software (BioRad, Hercules, CA) and defined as the ratio of target protein relative to Beta-actin. Antibodies for JARID2, p-AKT, AKT, vimentin, E-cadherin, PTEN and corresponding secondary antibodies were purchased from Santa Cruz Biotechnology (Santa Cruz Biotechnology, Santa Cruz, CA).

### Immunohistochemistry

Paraffin-embedded tissues were sectioned and microwave-pretreated in EDTA buffer (1mM, pH 8.0) for 10 minutes for antigen retrieval. Then, formalin-fixed paraffin sections were stained for JARID2 (Santa Cruz Biotechnology) using the streptavidin-peroxidase system (Zhong-shan Goldenbridge Biotechnology, Beijing, China). To support the validity of the immunohistochemistry, HCCLM3^shcontrol,^ HCCLM3^shJARID2-1^ cells were chosen used to perform cell immunohistochemistry to demonstrate the effectiveness of the antibodies JARID2 used, as PCR and Western blot showed HCCLM3 cells exhibited high JARID2 expression, and HCCLM3 transfected with the shJARID2-1 plasmid showed dramatically reduced JARID2 expression. Positive, negative control slides were probed with JARID2 antibody, goat serum respectively, and followed by the secondary antibody under the same conditions (Supplementary Figure 10A and 10B). The results proved that JARID2 antibody used was useful and specific for JARID2 (Supplementary Figure 10A 10B). The expression levels of JARID2 were scored using a four-point scale according to the percentage of positive hepatocytes: [40, 41] 0, ≤ 10% positive; 1+, 11%–25% positive; 2+, 26%–50% positive; 3+, ≥ 51% positive. The protein expression of JARID2 was thus considered negative if scored 0, and 1+, 2+, and 3+ as positive. According to the score of JARID2 expression, HCC specimens was also divided into a low expression group (0 or 1+) and a high expression group (2+ or 3+). For PTEN, p-AKT, vimentin, E-cadherin, the procedures as JARID2 and immunostaining score were adopt as described elsewhere [42]. Antibodies for PTEN, p-AKT, vimentin, E-cadherin were all purchased from Santa Cruz Biotechnology (Santa Cruz, CA).

### Cell proliferation and colony formation assays

For cell proliferation assay, 3 × 10^4^ HCC cells expressing a short hairpin targeting JARID2 or the control hairpin were seeded into a 35mm dishes (Corning Costar Corp, Corning, NY) in triplicate and cultured in 5% CO_2_ at 37°C. The cells were trypsinized and the number of cells was counted by using cell counter for seven consecutive days. For colony formation assays, 500 cells were seeded into 35 mm dishes (Corning Costar Corp) and cultured in 5% CO_2_ for 2 weeks at 37°C. The number of colonies per dish was counted after staining with crystal violet. Only positive colonies (diameter > 40 um) in the dishes were counted and compared [29, 43]. These experiments were performed in triplicate.

### Wound healing assays

Wound healing assays were performed and analyzed as described elsewhere [44]. Cells were seeded into six-well culture plates (Corning Costar Corp) coated with fibronectin. After cells reached 100% confluence, to suppress cell proliferation which could confound the analysis of cell migration, cells were preincubated with mitomycin (Sigma, St. Louis, MO, 10 μg/ml) for 1h at 37°C. Wound healing assays were performed with a sterile pipette tip to make a scratch through the confluent monolayer. Medium was changed and cells were cultured for 24 hours. The percent wound closure was calculated for three randomly chosen fields.

### Transwell assays

For the transwell assays, about 1 × 10^5^ cells in serum-free medium were placed into the upper chamber of the insert with matrigel plated (BD Biosciences, Franklin Lakes, NJ). After 24 hours of incubation in 5% CO_2_ at 37°C, the cells in upper chamber were removed with cotton swabs, following fixed by 20% methanol, and then stained with a solution containing 0.1% crystal violet (Beyotime Institute of Biotechnology, Beijing, China). The number of cells that adhered to the lower membrane of the inserts was counted. For each experimental group, the assays were performed in triplicates, and five random fields were chosen for analysis.

### Immunofluorescence

Immunofluorescence was performed as described previously [45]. Cells were seeded into the 6-well culture plate (Corning Costar Corp) to prepare for performing cell immunofluorescence (IF). After incubating with primary antibodies, cells then incubated with corresponding fluorescence labeled secondary antibody. The slides were photographed using the inverted fluorescence microscope TE-2000S (Nikon, Tokyo, Japan). Primary antibodies for E-cadherin, vimentin were purchased from Santa Cruz Biotechnology (Santa Cruz, CA). Rhodamine-conjugated phalloidin, DAPI and fluorescence labeled secondary antibody were obtained from Beyotime Institute of Biotechnology (Shanghai, China).

### Co-immunoprecipitation

Co-immunoprecipitation was performed as described previously [46]. For Co-immunoprecipitation assays, the cells were lysed with cold lysis buffer at 4°C for 30 min. Supernatants of cell lysates were then collected by centrifugation at 50,000 rpm for 10min, followed by incubation with an appropriate primary antibody overnight at 4°C. The immunocomplexes were next precipitated by protein A/G sepharose beads. After washes, the protein complexes were eluted out by sample buffer and then subjected to western blot analysis by probing with antibodies against EZH2 (Santa Cruz Biotechnology), SUZ12 (BD Biosciences), RbAp46/48 (BD Biosciences), EED (BD Biosciences) and JARID2 (Santa Cruz Biotechnology).

### Chromatin immunoprecipitation (ChIP) assays

ChIP was performed using a ChIP assay kit (Millipore, Billerica, MA) according to manufacturer's instructions. Briefly, cells were cross-linked with 1% formaldehyde. The chromatin was sonicated into fragments ranging between 200 and 1000 bp and then was pulled down by antibody for real-time quantitative PCR amplification. The antibodies used were anti-JARID2 (Santa Cruz), anti-H3K27me3 (Santa Cruz). The primers for amplifying the fragments of the PTEN promoter are as follows: 5′-CCGTGCATTTCCCTCTACAC-3′ (sense) and 5′- GAGGCGAGGATAACGAGCTA-3′ (antisense).

### *In vivo* assays for metastasis

For the *in vivo* metastasis assays, the HCC metastatic model in mice was constructed as previously described [29]. Briefly, 5 × 10^6^ HCC cells were injected subcutaneously into the left upper flank regions of nude mouse (4 weeks of age, male, BALB/c). After 35 days, the subcutaneous tumors were removed and divided into commensurate fragments of approximately 1 mm^3^, then implanted into the liver of nude mouse (3 mice in each group). After 6 weeks, mice were killed, and all livers and lungs were harvested. The tumor size was calculated as follows: tumor volume (mm^3^) = (L × W^2^)/2, [47] where L = long axis and W = short axis. All livers and lungs were fixed with 10% phosphate-buffered neutral formalin, sectioned serially and stained with hematoxylin and eosin (H&E) for histological examination. The expression of JARID2, PTEN, p-AKT, vimentin, E-cadherin in orthotropic tumor tissues was also determined by immunohistochemistry. Mice were performed and housed in the Animal Institute of CSU according to the protocols approved by the Medical Experimental Animal Care Commission.

### Statistical analysis

All data were analyzed using the statistical software SPSS 18.0 for Windows (SPSS Inc., Chicago, IL). The differences between groups were analyzed by Student's *t* test between two groups or by one-way analysis of variance (ANOVA) in more than two groups when the variance is homogeneous. If the variance is not homogeneous, the differences between groups were analyzed by Mann-Whitney *U* test between two groups or by Kruskal-Wallis *H* test in more than two groups. *χ*^2^ analysis was used to analyze the correlation between JARID2 expression and clinicopathologic features. Linear regression was used to analyze the relationship between JARID2 and PTEN mRNA expression. Spearman's rank analysis was used to analyze the correlations between different protein expressions level. Survival curves were constructed using the Kaplan-Meier method and evaluated using the log-rank test. The Cox proportional hazards regression model was established to identify factors which were independently associated with the overall survival (OS) and disease-free survival (DFS) of HCC patients. All the tests were two-tailed and *P* < 0.05 was considered statistically significant.

## SUPPLEMENTARY MATERIALS FIGURES AND TABLES






