# Supplementary material for: JARID2 promotes invasion and metastasis of hepatocellular carcinoma by facilitating epithelial-mesenchymal transition through PTEN/AKT signaling
Source: Oncotarget. 2016 May 31;7(26):40266–84. doi: 10.18632/oncotarget.9733 (PMC5130007; doi:10.18632/oncotarget.9733)
Supplement: Supplementary file 1 [file oncotarget-07-40266-s001.pdf]

# JARID2 promotes invasion and metastasis of hepatocellular carcinoma by facilitating epithelial-mesenchymal transition through PTEN/AKT signaling

## Supplementary Materials

**Supplementary Table S1: Clinicopathologic characteristics of the HCC patients in training cohort and validation cohort.** See Supplementary_Table_S1

**Supplementary Table S2: Correlations between JARID2 expression in the adjacent non-tumor liver tissues (ANLT) and clinicopathologic variables of HCC in training and validation cohort.** See Supplementary Table S2

**Supplementary Table S3: Correlation between JARID2 expression and PTEN, p-AKT, E-cadherin, vimentin expression in HCC tissues from training and validation cohorts**

| | Training cohort | | | | Validation cohort | | | |
|---|---|---|---|---|---|---|---|---|
| | *n* | JARID2 expression level | | *P* value | *n* | JARID2 expression level | | *P* value |
| | | Low (*n* = 47) | High (*n* = 69) | *r* | | Low (*n* = 28) | High (*n* = 38) | *r* |
| **PTEN expression level** | | | | | | | | |
| Low | 80 | 23 | 57 | **< 0.001** | 40 | 13 | 27 | **0.044** |
| High | 36 | 24 | 12 | **−0.357** | 26 | 15 | 11 | **−0.249** |
| **p-AKT expression level** | | | | | | | | |
| Low | 46 | 25 | 21 | **0.014** | 27 | 18 | 9 | **0.001** |
| High | 70 | 22 | 48 | **0.228** | 39 | 10 | 29 | **0.408** |
| **E-cadherin expression level** | | | | | | | | |
| Low | 66 | 21 | 45 | **0.028** | 37 | 11 | 26 | **0.018** |
| High | 50 | 26 | 24 | **−0.204** | 29 | 17 | 12 | **−0.290** |
| **Vimentin expression level** | | | | | | | | |
| Low | 49 | 27 | 22 | **0.006** | 32 | 19 | 13 | **0.007** |
| High | 67 | 20 | 47 | **0.254** | 34 | 9 | 25 | **0.333** |

**Supplementary Table S4: Correlation between PTEN expression and p-AKT, E-cadherin, vimentin expression in HCC tissues from training and validation cohorts**

| | Training cohort | | | | Validation cohort | | | |
|---|---|---|---|---|---|---|---|---|
| | *n* | PTEN expression level | | *P* value | *n* | PTEN expression level | | *P* value |
| | | Low (*n* = 80) | High (*n* = 36) | *r* | | Low (*n* = 40) | High (*n* = 26) | *r* |
| **p-AKT expression level** | | | | | | | | |
| Low | 46 | 20 | 26 | **< 0.001** | 27 | 11 | 16 | **0.005** |
| High | 70 | 60 | 10 | **−0.447** | 39 | 29 | 10 | **−0.338** |
| **E-cadherin expression level** | | | | | | | | |
| Low | 66 | 53 | 13 | **0.002** | 37 | 30 | 7 | **< 0.001** |
| High | 50 | 27 | 23 | **0.282** | 29 | 10 | 19 | **0.473** |
| **Vimentin expression level** | | | | | | | | |
| Low | 49 | 25 | 24 | **< 0.001** | 32 | 12 | 20 | **< 0.001** |
| High | 67 | 55 | 12 | **−0.332** | 34 | 28 | 6 | **−0.459** |

**Supplementary Table S5: The sequences of shRNA used in this study**

| Name | Sense sequence |
|---|---|
| JARID2-shRNA-sequence-1 | 5′-CCGGGAAACAGGTTTCTAAGGTAAACTCGAGTTTACCTTAGAAACCTGTTTCTTTTTG-3′ |
| JARID2-shRNA-sequence-2 | 5′-CCGGGAGGGCTGAAGTTGATGTATTCTCGAGAATACATCAACTTCAGCCCTATTTTTG-3′ |
| JARID2-shRNA-sequence-3 | 5′-CCGGGCCCAACAGCATGGTGTATTTCTCGAGAAATACACCATGCTGTTGGGCTTTTTG-3′ |
| JARID2-shRNA-sequence-4 | 5′-CCGGCGCTACGATGAGGAACAGATTCTCGAGAATCTGTTCCTCATCGTAGCGTTTTTG-3′ |
| JARID2-shRNA-sequence-5 | 5′-CCGGCCTCCACTAGCAACGATGTTACTCGAGTAACATCGTTGCTAGTGGAGGTTTTTG-3′ |
| PTEN-shRNA-sequence-1 | 5′-CCGGCCACAGCTAGAACTTATCAAACTCGAGTTTGATAAGTTCTAGCTGTGGTTTTTG-3′ |
| PTEN-shRNA-sequence-2 | 5′-CCGGAGGCGCTATGTGTATTATTATCTCGAGATAATAATACACATAGCGCCTTTTTTG-3′ |
| PTEN-shRNA-sequence-3 | 5′-CCGGGGGCTTTAACTGTAGTATTTGCTCGAGCAAATACTACAGTTAAAGCCCTTTTTG-3′ |
| Control sequence | 5′-CCGGAGCGTTCACTCCCAACCTGCTCGAGCAGGTTGGGAGTGAACGCTTTTTTG-3′ |

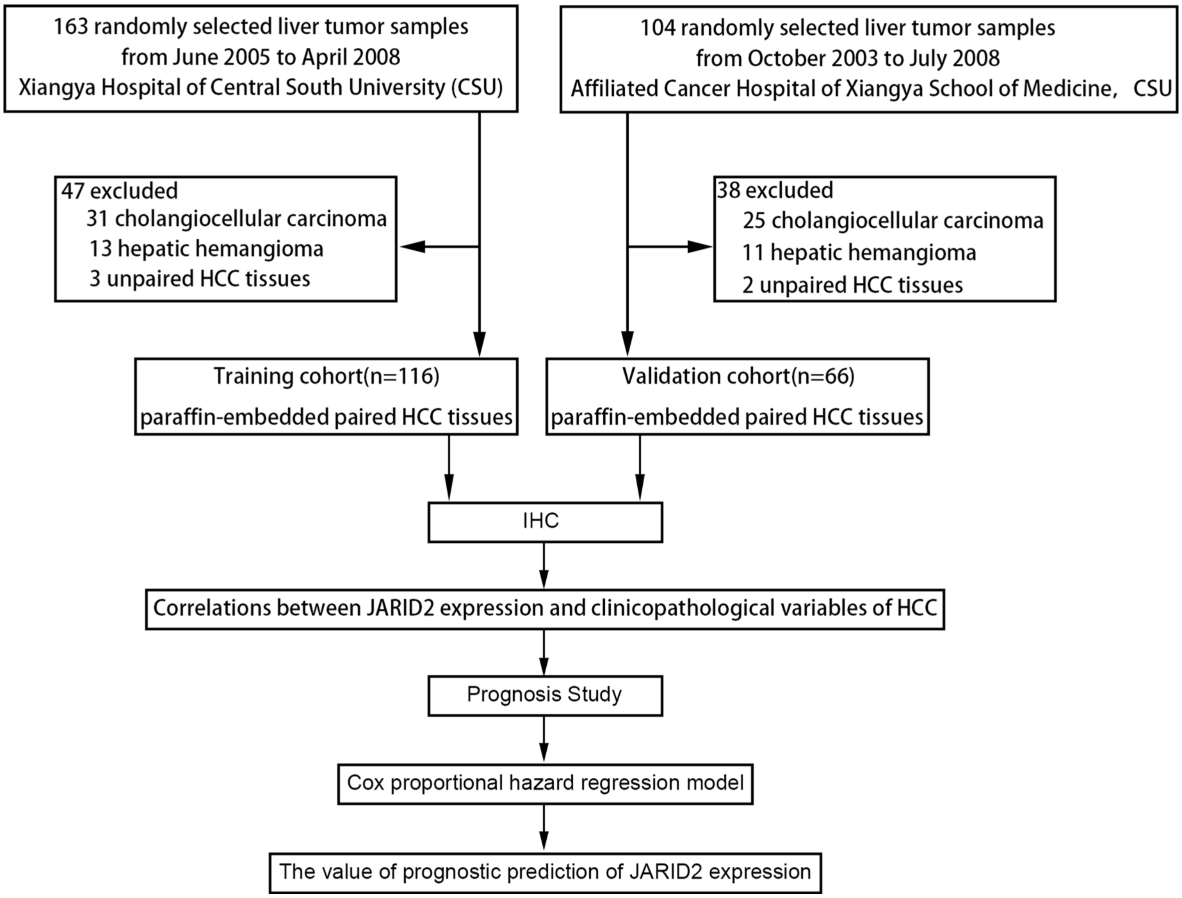


**Supplementary Figure S1: A flowchart of clinical experimental design in two independent cohorts of HCC patients for prognostic study.**

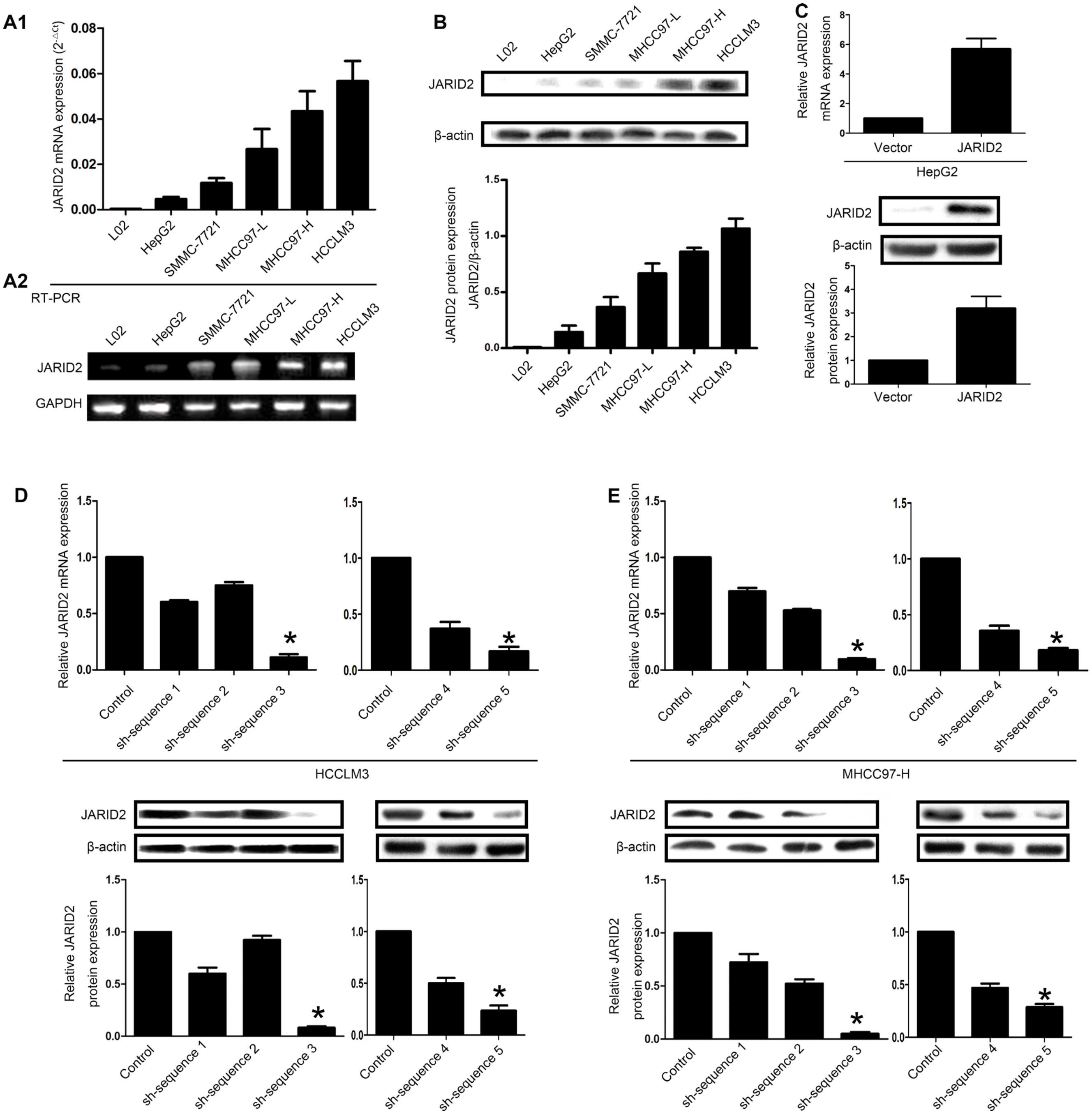


**Supplementary Figure S2: JARID2 expression in HCC cell lines.** (**A**) JARID2 mRNA expression level was detected by quantitative real-time polymerase chain reaction (qRT-PCR) (**A1**) and semiquantitative RT-PCR (**A2**) in five HCC cell lines and normal cell line L02. The results showed that, compared to L02 cells, HCCLM3 cells have the highest JARID2 expression level, followed by MHCC97-H, MHCC97-L, SMMC-7721 and HepG2 cells. (**B**) JARID2 protein expression level was detected by western blot in five HCC cell lines and normal cell line L02. The results showed that, compared to L02 cells, HCCLM3 cells have the highest JARID2 expression level, followed by MHCC97-H, MHCC97-L, SMMC-7721 and HepG2 cells. (**C**) JARID2-expression plasmid upregulates JARID2 expression in HepG2 cells. qRT-PCR and western blot were employed to evaluate the upregulation of exogenous JARID2 expression in HepG2 cells. (**D** and **E**) Downregulation of JARID2 expression via shRNA-JARID2 plasmid in HCCLM3 cells (D) and MHCC97-H cells (E). qRT-PCR and western blot was used to check the inhibitory efficiency of five designed JARID2 shRNAs and control. The expression of JARID2 in control cell was set to 1. The relative expression levels of JARID2 in these cells were displayed in the diagram. The JARID2-shRNA-Seq3 (named shJARID2-1) reduced JARID2 expression above 90% and JARID2-shRNA-Seq5 (named shJARID2-2) reduced JARID2 expression above 70%. These two JARID2 shRNAs was adopted for subsequent study because of its highly effective inhibitory efficiency.

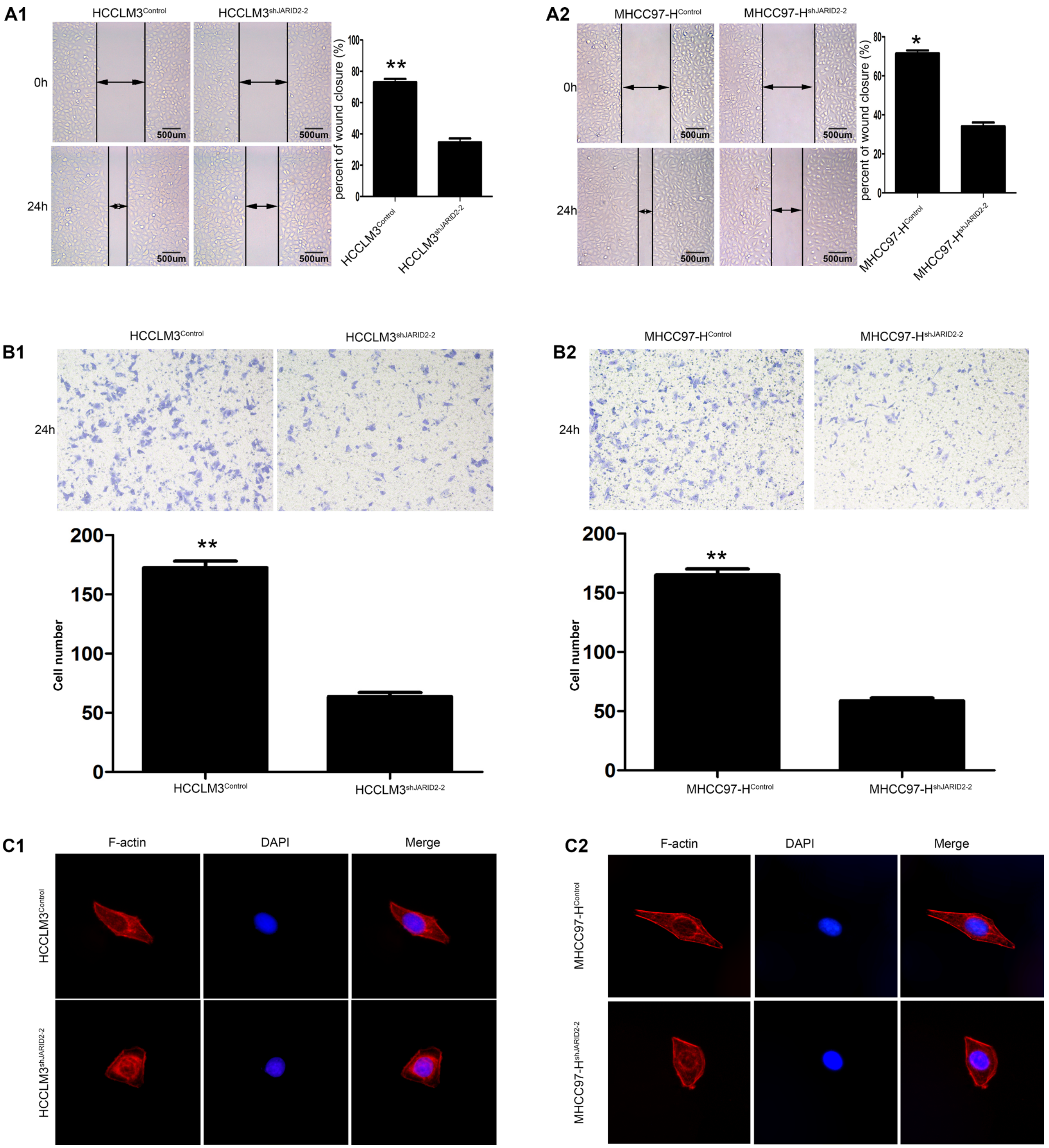


**Supplementary Figure S3: Knockdown JARID2 expression significantly inhibited migration and invasion of HCC cells *in vitro*.** (**A**) Knockdown of JARID2 expression with shJARID2-Seq5 (named shJARID-2 in the Figure) inhibited HCCLM3 (**A1**) and MHCC97-H (**A2**) cells migration in wound-healing assays. (**B**) The transwell assays showed that knockdown of JARID2 expression with shJARID-2 inhibited HCCLM3 (**B1**) and MHCC97-H (**B2**) cells invasion. (**C**) Immunofluorescence assays of cytoskeleton of $HCCLM3^{control}$ and $HCCLM3^{shJARID2-2}$ (**C1**), $MHCC97\text{-}H^{control}$, $MHCC97\text{-}H^{shJARID2-2}$cells (**C2**). F-actin filaments were visualized in cells using rhodamine-phalloidin.

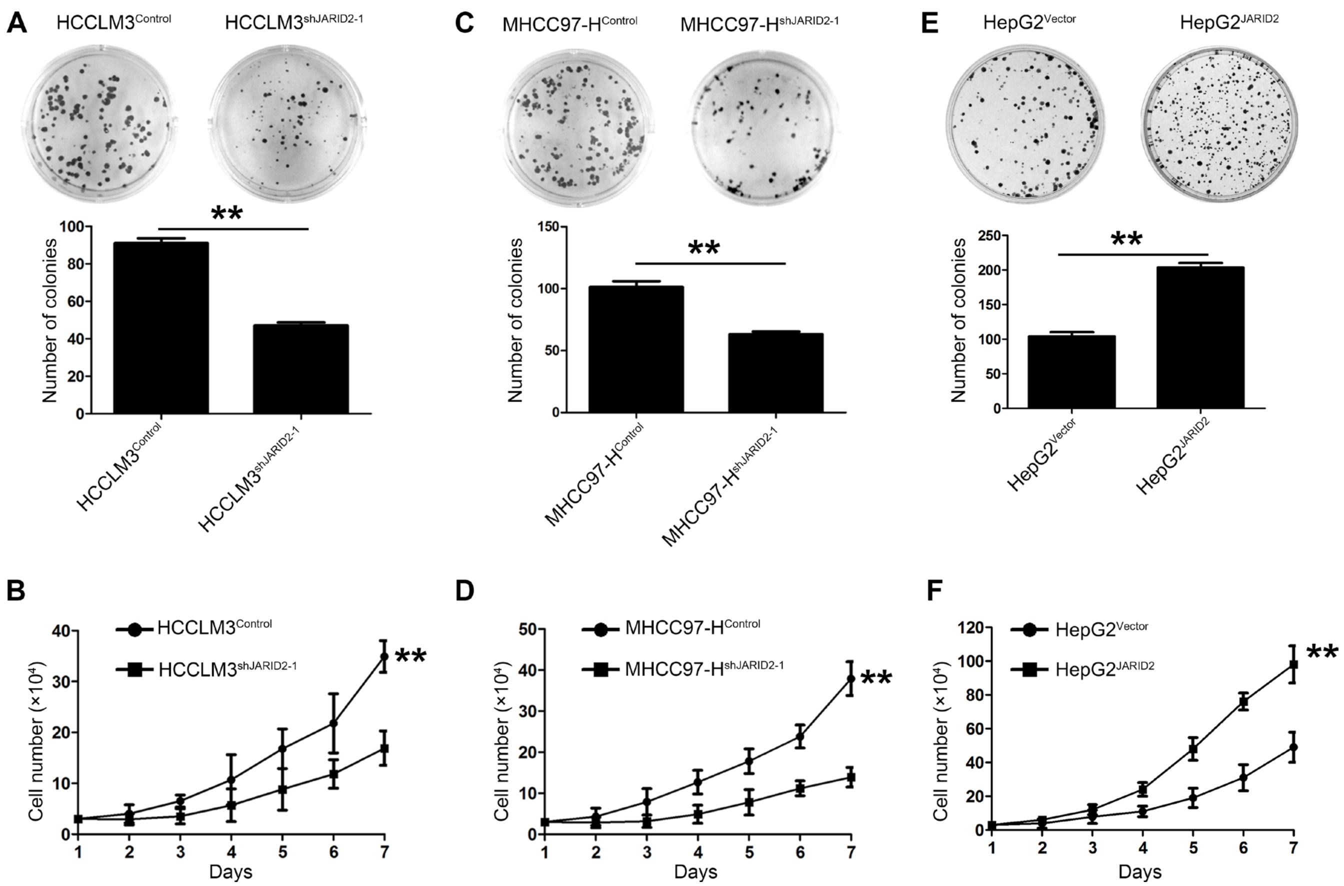


**Supplementary Figure S4: JARID2 regulates HCC cells proliferation.** (**A** and **C**) Colony formation assays were performed to assess the effect of JARID2 on HCCLM3 (A), MHCC97-H (C), HepG2 cells (**E**) proliferation. Knockdown JARID2 in HCCLM3 (A), MHCC97-H (C) cells with shJARID2-1 significantly inhibited colony formation, whereas JARID2 overexpression in HepG2 cells promoted colony formation. The number of colonies was counted and compared in the diagrams. (**B**, **D**, **F**) Growth curve was used to determine the proliferation of HCCLM3 (B), MHCC97-H (D), HepG2 cells (F). $^{**}P < 0.01$

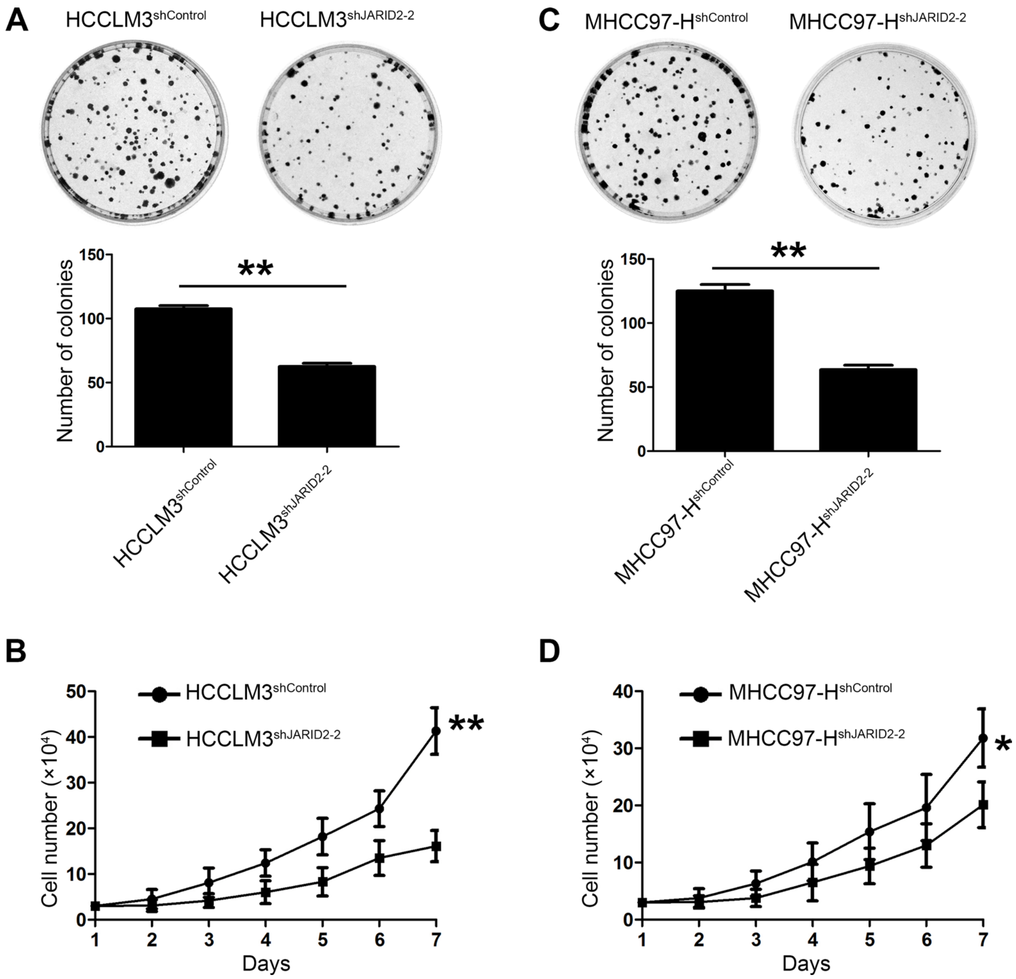


**Supplementary Figure S5: Knockdown JARID2 expression significantly inhibited HCC cells proliferation.** (**A** and **C**) Knockdown JARID2 in HCCLM3 (A), MHCC97-H (C) cells with shJARID2-2 significantly inhibited colony formation. The number of colonies was counted and compared in the diagrams. (**B** and **D**) Growth curve was used to determine the proliferation of HCCLM3 (B), MHCC97-H (D). **P* < 0.05; ***P* < 0.01.

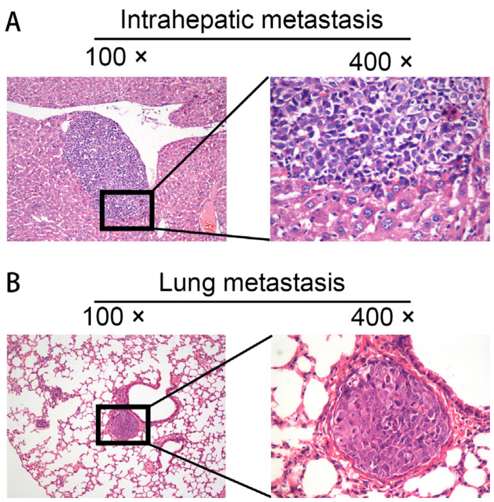


**Supplementary Figure S6, related to Figure 3F3, SF4: (A and B) Representative pictures for intrahepatic (A) and lung metastasis (B) from mice transplanted with HepG2$^{Vector}$ and HepG2$^{shJARID2}$ cells.**

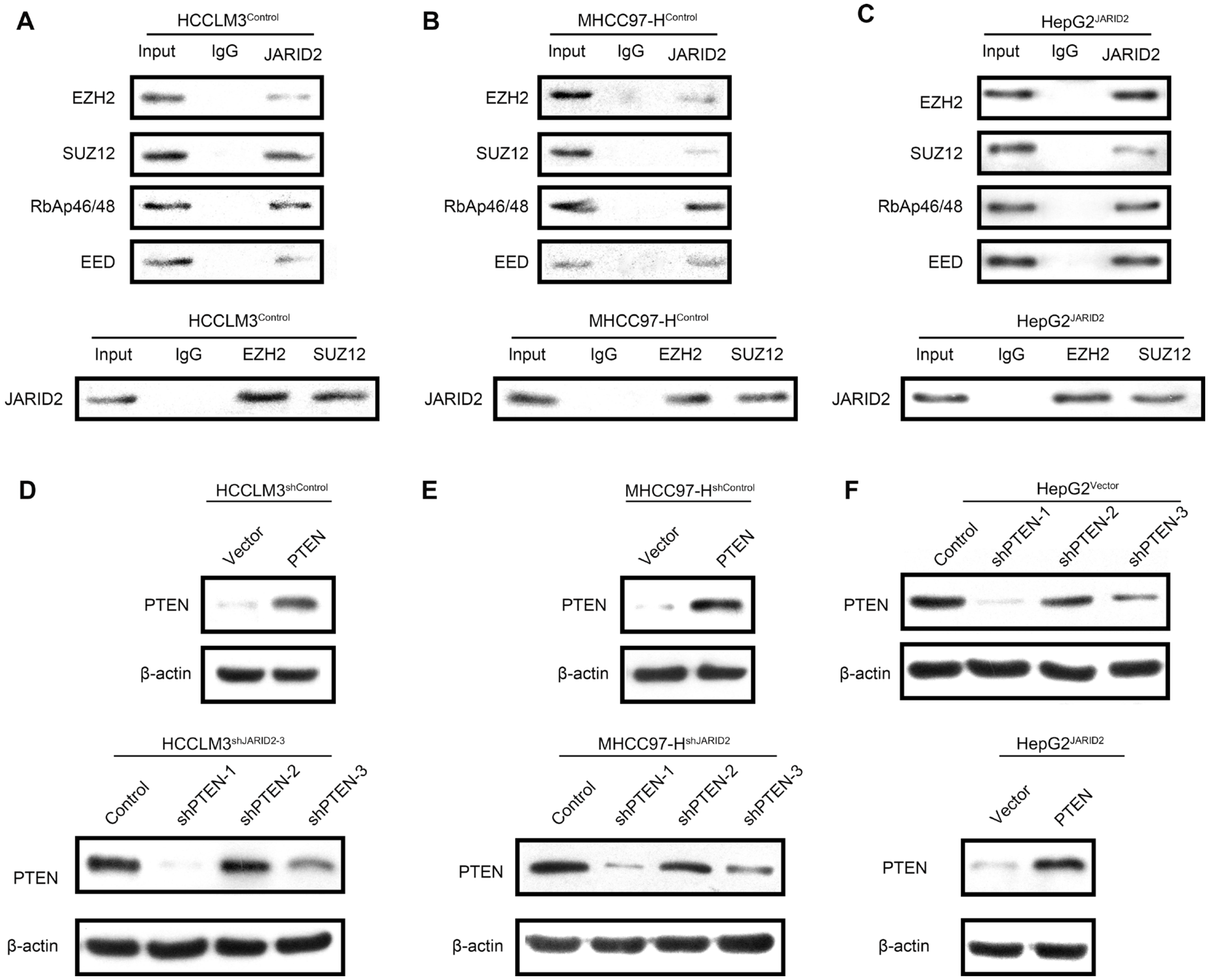


**Supplementary Figure S7:** (**A** and **B**) JARID2 interacts with PRC2 in HCC cells. Association of JARID2 with PRC2 was analyzed in HCCLM3 cells (A), MHCC97-H cells (B), and HepG2 cells (**C**). Whole cell lysates were immunoprecipitated with the specific antibodies against the indicated proteins. Immunocomplexes in the precipitates were then immunoblotted using antibodies against the indicated proteins. Antibodies specific for JARID2, Suz12, Ezh2 and RbAp46/48 were used for immunoprecipitations (IPs); 5% of input is presented as loading control. IgG served as the negative control. (**D**, **E**, **F**) Western blot was used to determine the inhibitory efficiency of PTEN or ectopic PTEN expression in HCCLM3 cells (D), MHCC97-H cells (E), and HepG2 cells (F) transfected with lentivirus particles carrying specific shRNA for PTEN, PTEN expression vector and their corresponding control vector. The shRNA-1 (named shPTEN-1) and shRNA-3 (named shPTEN-3) reduced PTEN expression at least 80%. These two PTEN shRNAs were adopted for subsequent study because of its highly effective inhibitory efficiency.

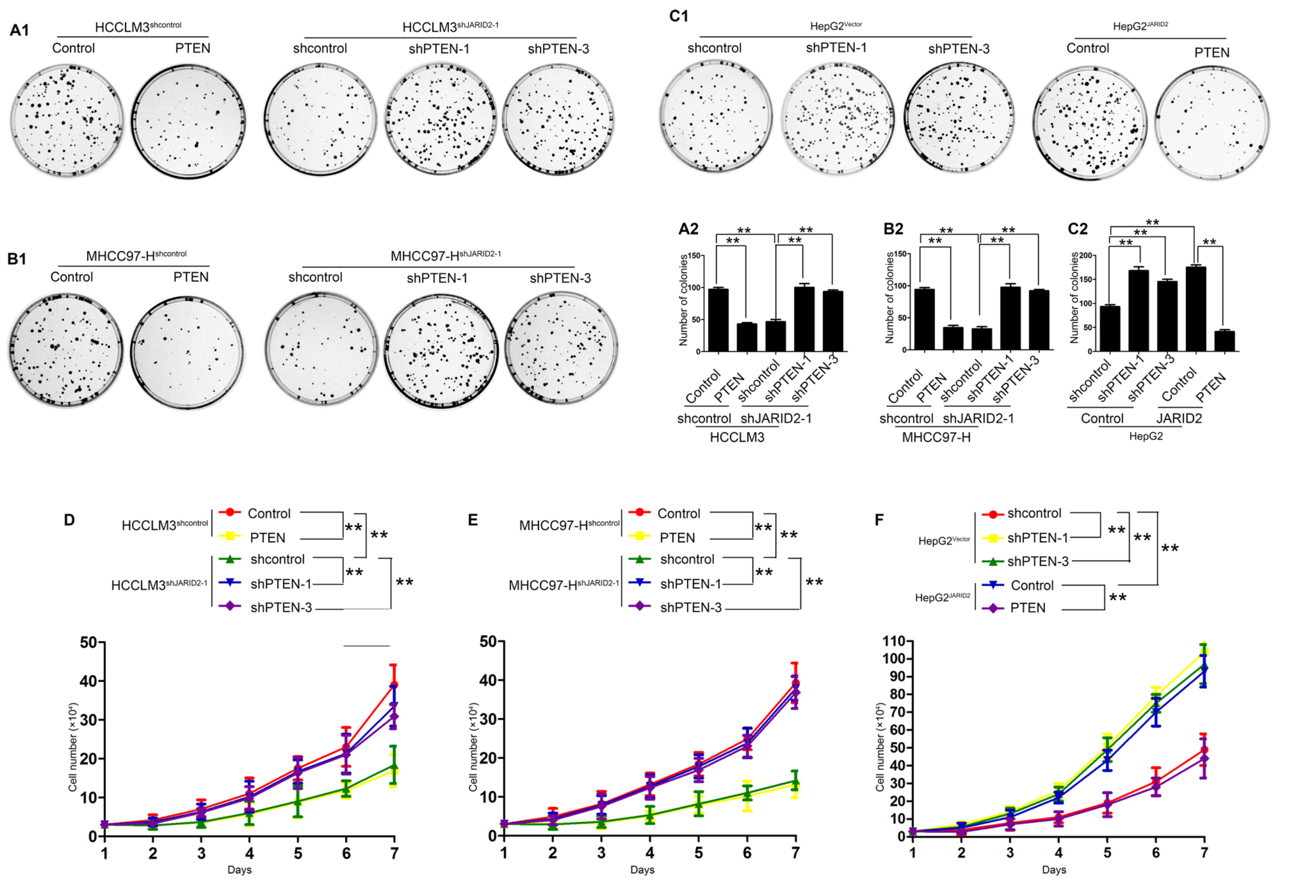


**Supplementary Figure S8: PTEN Is a critical mediator for JARID2-regulated HCC cells proliferation.** To investigate whether PTEN expression may attenuate or mimic the function of JARID2, HCC cells were re-transfected by shRNA for PTEN or PTEN expression vector to inhibit or restore the PTEN expression. (**A**) Colony formation assays showed overexpression of PTEN reduced the ability of colony formation of JARID2 expressing HCCLM3$^{shcontrol}$ (**A1**, **A2**), MHCC97-H$^{shcontrol}$ cells (**B1**, **B2**) and inhibition of PTEN expression also recovered the ability of the colony formation of HCCLM3$^{shJARID2-1}$ (**A1**, **A2**), MHCC97-H$^{shJARID2-1}$ cells (**B1**, **B2**), while silence of PTEN expression mimicked the effect of JARID2 on colony formation ability in HepG2 cells and overexpression of PTEN expression inhibited JARID2-mediated promotion of HepG2 cell colony formation ability (**C1**, **C2**). (**D**, **E**, **F**) cell growth curves were also performed to determine cell proliferation with above cells. $^{**}P < 0.01$.

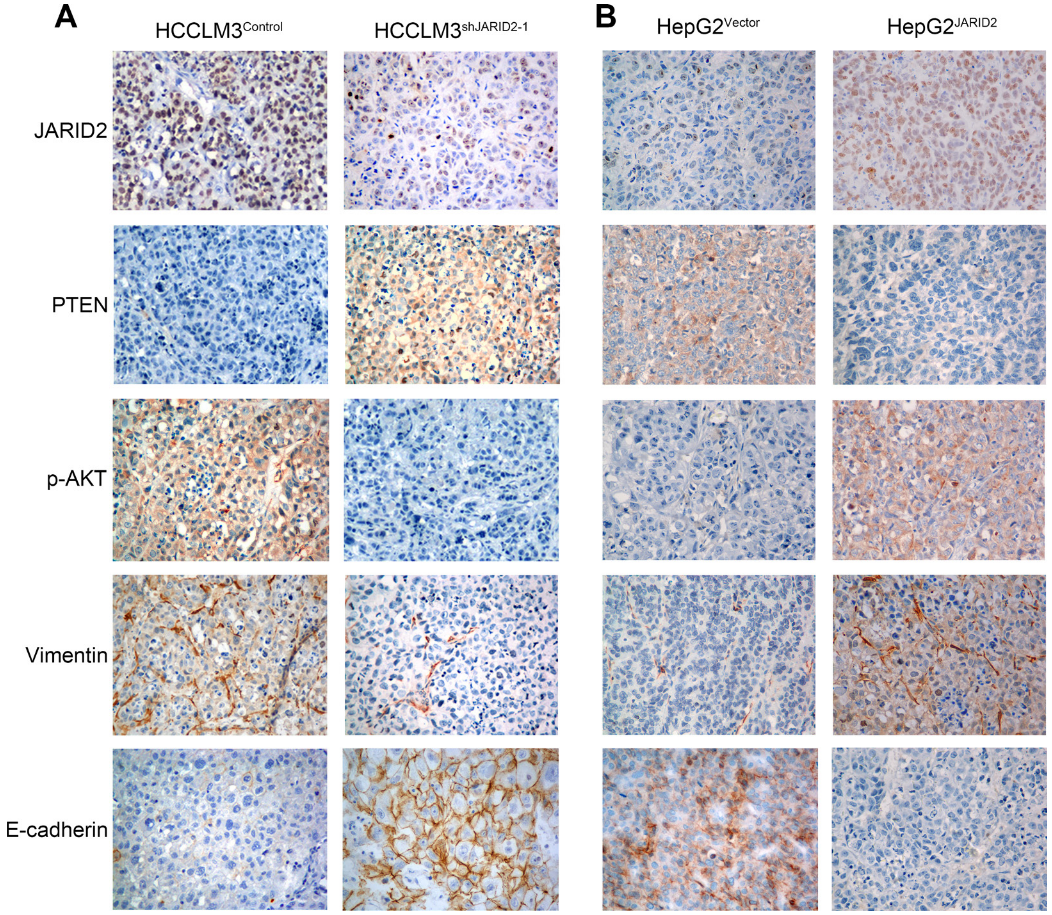


**Supplementary Figure S9:** (**A** and **B**) Representative Immunohistochemistry Staining Images of JARID2, PTEN, p-AKT, E-cadherin, and Vimentin from Orthotopic Tumor Sections Transplanted HCCLM3$^{shJARID2-1}$ and HCCLM3$^{Control}$ Cells (**A**), HepG2$^{Vector}$ and HepG2$^{JARID2}$ (**B**) (×400 magnification).

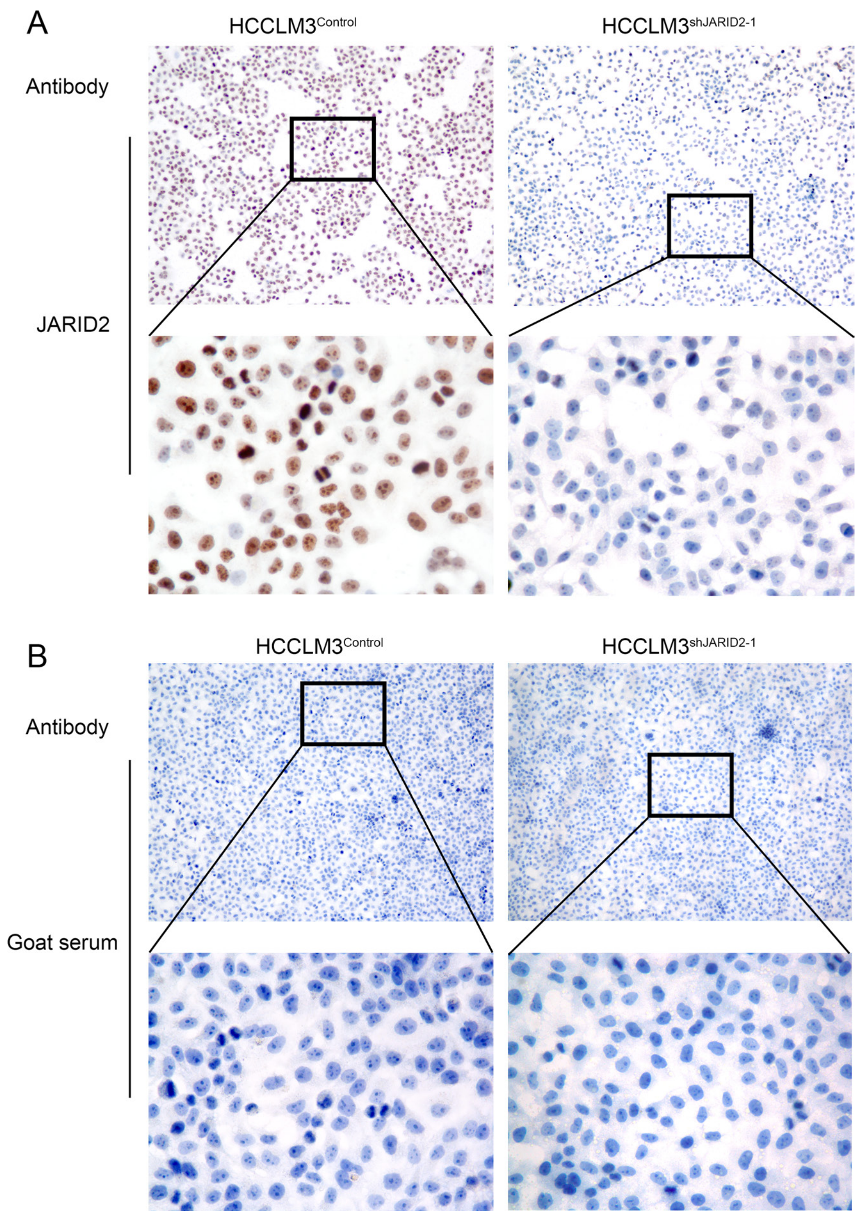


**Supplementary Figure S10: Cell Immunohistochemistry to support the validity of the immunohistochemistry and demonstrate the effectiveness of the antibodies JARID2 Used.** (**A**) HCCLM3$^{shcontrol}$, HCCLM3$^{shJARID2-1}$ slides probed with JARID2 antibody as primary antibody, and then followed by the secondary antibody were used for positive control. (**B**) The slides probed with goat serum as primary antibody, and then followed by the secondary antibody under the same conditions were used for negative controls.
