# Supplementary material for: JARID2 promotes invasion and metastasis of hepatocellular carcinoma by facilitating epithelial-mesenchymal transition through PTEN/AKT signaling
Source: Oncotarget. 2016 May 31;7(26):40266–84. doi: 10.18632/oncotarget.9733 (PMC5130007; doi:10.18632/oncotarget.9733)
Supplement: Supplementary file 2 [file oncotarget-07-40266-s002.docx]

**Supplementary Table S1: Clinicopathologic characteristics of the HCC patients in training cohort and validation cohort**

| Clinicopathologic Variables | Counts | | **P** value |
| --- | --- | --- | --- |
|  | Training cohort | Validation cohort |  |
| Gender |  |  |  |
| Female | 13 | 16 | 0.683 |
| Male | 103 | 50 |  |
| Age (years) |  |  |  |
| < 60 | 93 | 56 | 0.931 |
| ≥ 60 | 23 | 10 |  |
| AFP (ng/mL) |  |  |  |
| ≤ 20 | 56 | 23 | 0.079 |
| > 20 | 60 | 43 |  |
| HBsAg |  |  |  |
| Negative | 29 | 18 | 0.863 |
| Positive | 87 | 48 |  |
| Liver cirrhosis |  |  |  |
| Absent | 31 | 29 | 0.617 |
| Present | 85 | 37 |  |
| Child-Pugh classification |  |  |  |
| A | 102 | 59 | 0.807 |
| B | 14 | 7 |  |
| Tumor number |  |  |  |
| Solitary | 56 | 32 | 0.978 |
| Multiple (≥ 2) | 60 | 34 |  |
| Tumor size (cm) |  |  |  |
| ≤ 5 | 39 | 17 | 0.269 |
| > 5 | 77 | 49 |  |
| Capsular formation |  |  |  |
| Present | 52 | 36 | 0.207 |
| Absent | 64 | 30 |  |
| Microvascular invasion |  |  |  |
| Absence | 48 | 41 | 0.572 |
| Presence | 68 | 25 |  |
| Edmondson-Steiner grade |  |  |  |
| Low grade (I–II) | 36 | 30 | 0.372 |
| High grade (III–IV) | 80 | 36 |  |
| HCC subtype |  |  |  |
| SHCC | 24 | 14 | 0.996 |
| SLHCC | 32 | 18 |  |
| NHCC | 60 | 34 |  |
| TNM stage |  |  |  |
| I | 51 | 27 | 0.823 |
| II–III | 65 | 39 |  |
| BCLC stage |  |  |  |
| 0–A | 49 | 21 | 0.638 |
| B–C | 67 | 45 |  |

Abbreviations: AFP, alpha-fetoprotein; TNM, tumor node metastasis; BCLC, Barcelona Clinic Liver Cancer; SHCC, small hepatocellular carcinoma; SLHCC, solitary large hepatocellular carcinoma; NHCC, nodular hepatocellular carcinoma.
