# Supplementary material for: JARID2 promotes invasion and metastasis of hepatocellular carcinoma by facilitating epithelial-mesenchymal transition through PTEN/AKT signaling
Source: Oncotarget. 2016 May 31;7(26):40266–84. doi: 10.18632/oncotarget.9733 (PMC5130007; doi:10.18632/oncotarget.9733)
Supplement: Supplementary file 3 [file oncotarget-07-40266-s003.docx]

**Supplementary Table S2: Correlations between JARID2 expression in the adjacent non-tumor liver tissues (ANLT) and clinicopathologic variables of HCC in training and validation cohort**

| Clinicopathologic variable | Training Cohort | | | |  |  | Validation Cohort | |  |
| --- | --- | --- | --- | --- | --- | --- | --- | --- | --- |
|  | No. | JARID2 expression levels | |  |  | No. | JARID2 expression levels | |  |
|  |  | Low (n = 101) | High (n = 15) | P value |  |  | Low (n = 64) | High (n = 2) | P value |
| Gender |  |  |  |  |  |  |  |  |  |
| Female | 13 | 10 | 3 | 0.473 |  | 16 | 16 | 0 | 0.571 |
| Male | 103 | 91 | 12 |  |  | 50 | 48 | 2 |  |
| Age (years) |  |  |  |  |  |  |  |  |  |
| ≤ 60 | 93 | 82 | 11 | 0.715 |  | 56 | 55 | 1 | 0.282 |
| > 60 | 23 | 19 | 4 |  |  | 10 | 9 | 1 |  |
| AFP(ng/mL) |  |  |  |  |  |  |  |  |  |
| ≤ 20 | 56 | 49 | 7 | 0.894 |  | 23 | 23 | 0 | 0.421 |
| > 20 | 60 | 52 | 8 |  |  | 43 | 41 | 2 |  |
| HBsAg |  |  |  |  |  |  |  |  |  |
| Negative | 29 | 24 | 5 | 0.632 |  | 18 | 17 | 1 | 0.474 |
| Positive | 87 | 77 | 10 |  |  | 48 | 47 | 1 |  |
| Liver cirrhosis |  |  |  |  |  |  |  |  |  |
| Absence | 31 | 25 | 6 | 0.351 |  | 29 | 28 | 1 | 0.690 |
| Presence | 85 | 76 | 9 |  |  | 37 | 36 | 1 |  |
| Child-Pugh classification |  |  |  |  |  |  |  |  |  |
| A | 102 | 89 | 13 | 0.872 |  | 59 | 57 | 2 | 0.798 |
| B | 14 | 12 | 2 |  |  | 7 | 7 | 0 |  |
| Tumor number |  |  |  |  |  |  |  |  |  |
| Solitary | 56 | 48 | 8 | 0.674 |  | 32 | 31 | 1 | 0.738 |
| Multiple | 60 | 53 | 7 |  |  | 34 | 33 | 1 |  |
| Tumor size |  |  |  |  |  |  |  |  |  |
| ≤ 5 cm | 39 | 33 | 6 | 0.575 |  | 17 | 17 | 0 | 0.548 |
| > 5 cm | 77 | 68 | 9 |  |  | 49 | 47 | 2 |  |
| Capsular formation |  |  |  |  |  |  |  |  |  |
| Presence | 52 | 46 | 6 | 0.687 |  | 36 | 35 | 1 | 0.706 |
| Absence | 64 | 55 | 9 |  |  | 30 | 29 | 1 |  |
| Microvascular invasion |  |  |  |  |  |  |  |  |  |
| Absence | 68 | 60 | 8 | 0.656 |  | 41 | 39 | 2 | 0.382 |
| Presence | 48 | 41 | 7 |  |  | 25 | 25 | 0 |  |
| Edmondson-Steiner grade |  |  |  |  |  |  |  |  |  |
| Low grade (I and II) | 36 | 32 | 4 | 0.926 |  | 30 | 29 | 0 | 0.310 |
| High grade (III and IV) | 80 | 69 | 11 |  |  | 36 | 35 | 2 |  |
| HCC subtype |  |  |  | 0.618# |  |  |  |  | 0.428# |
| SHCC | 24 | 20 | 4 | 1.000## |  | 14 | 13 | 1 | 0.438## |
| SLHCC | 32 | 27 | 5 | 0.632* |  | 18 | 18 | 0 | 0.503* |
| NHCC | 60 | 54 | 6 | 0.649** |  | 34 | 33 | 1 | 0.654** |
| TNM Stage |  |  |  |  |  |  |  |  |  |
| I | 51 | 45 | 6 | 0.740 |  | 27 | 27 | 0 | 0.345 |
| II–III | 65 | 56 | 9 |  |  | 39 | 37 | 2 |  |
| BCLC Stage |  |  |  |  |  |  |  |  |  |
| 0–A | 49 | 42 | 7 | 0.710 |  | 21 | 21 | 0 | 0.462 |
| B–C | 67 | 59 | 8 |  |  | 45 | 43 | 2 |  |

Abbreviations: AFP, alpha-fetoprotein; TNM, tumor node metastasis; BCLC, Barcelona Clinic Liver Cancer; SHCC, small hepatocellular carcinoma; SLHCC, solitary large hepatocellular carcinoma; NHCC, nodular hepatocellular carcinoma; #, SHCC vs. SLHCC vs. NHCC; ^##^SHCC vs. SLHCC; *SHCC vs. NHCC; **SLHCC vs. NHCC.
